# Comparative analysis of four complete mitogenomes from hoverfly genus *Eristalinus* with phylogenetic implications

**DOI:** 10.1038/s41598-022-08172-6

**Published:** 2022-03-09

**Authors:** Hu Li, Juan Li

**Affiliations:** Shaanxi Key Laboratory of Bio-Resources, School of Biological Science & Engineering, Shaanxi University of Technology, Hanzhong, 723000 Shaanxi China

**Keywords:** Evolutionary genetics, Phylogenetics, Genome, Genomics, Entomology

## Abstract

The genus *Eristalinus* is widely distributed globally. Four complete mitochondrial genomes (i.e., mitogenomes) of *Eristalinus* were sequenced and analyzed in this study: *Eristalinus viridis* (Coquillett, 1898), *E. quinquestriatus* (Fabricius, 1781), *E. tarsalis* (Macquart, 1855), and *E.* sp*.* Within these four sequenced mitogenomes, most protein-coding genes (*ND2*, *CO1*, *COX2*, *COX3*, *ND3*, *ND5*, *ND4*, *ND4L*, *ND6*, and *Cytb*) began with a typical ATN (T/C/G/A) start codon and ended with a stop codon TAA or incomplete T, whereas *ND1* began with the start codon TTG. *ND3* ended with TAG. The secondary tRNA structure was that of a typical cloverleaf, and only the *tRNA-Ser1* lacked a DHU arm. Three and five domains appeared in the *12S* and *16S* rRNA secondary structures, respectively. The phylogenetic relationships among the four *Eristalinus* species combined with the published mitogenomes of Syrphidae were reconstructed using the maximum likelihood and Bayesian inference methods, which support the monophyly of the subfamily Syrphinae but do not support that of the subfamily Eristalinae. Of note, Eristalini and Syrphini are monophyletic groups. The mitogenomes of *E. viridis*, *E. quinquestriatus*, *E.* sp*.*, and *E. tarsalis* are useful for determining the phylogenetic relationships and evolution of Syrphidae.

## Introduction

Syrphidae is a large family with high species diversity within the order Diptera, with more than 6000 species^1,2^ distributed worldwide. They are well recognized because most have bright black and yellow patterns on the abdomen and are similar to bumblebees, wasps, or honeybees; this mimicry can help syrphids escape from their natural enemies^3^. Different researchers follow different systems with respect to their higher taxonomy classification^4,5^. The most population is the three subfamilies system, Syrphinae, Eristalinae, and Microdontinae^5^. However, recent studies suggest a four subfamilies system: Syrphinae, Eristalinae, Microdontinae, and Pipizinae^6,7^. Based on mtDNA sequences and morphology, Eristalinae has been deemed as paraphyletic group^8^. However, Eristalinae has been deemed as monophyletic group based on sequence data of nuclear *28S* rRNA and mitochondrial cytochrome oxidase c subunit 1 (*COI*) genes in conjunction with larval and adult morphological characteristics of syrphid taxa^9^.

The mitochondrial genome is a circular and double-stranded DNA molecule, with a low molecular (14–36 kb), rapid evolution rate, and stable gene composition. Many insect groups utilize their mitochondrial sequences to solve phylogenetic problems ^10–13^. Thus far, 20 complete mitogenomes of Syrphidae have been sequenced and uploaded to GenBank. Sonet et al.^14^ published five Afrotropical species of *Eristalinus* (*E. barclayi*, *E. fuscicornis*, *E. vicarians*, *E. aeneus* and *E. tabanoides*) and attempted to resolve the phylogenetic relationships of *Eristalinus* from phylogenetic tree analysis and informativeness of 13 protein-coding genes (PCGs) and 2 rRNAs. More molecular data could help establish Syrphidae in a stable classification system and aid in further understanding its evolutionary history.

In previous studies on mitogenomes, Syrphinae was described as a monophyletic group^14–19^ and the tribes Syrphini and Melanostomini as sister groups^19,20^. Eristalinae has not been established as a monophyletic group; moreover, Volucellini, Cheilosiini, and Milesiini are strongly related groups, and together clustered a clade as a sister group to the Syrphinae. Eristalini is a cluster, but relation of the genera under Eristalini needs to be further discussed^14,21^.

*Eristalinus* Rondani, 1845 (Diptera: Syrphidae, Eristalinae) is widely distributed worldwide. This genus contains approximately 75 species, with at least 15 distributed in China. Adults typically visit flowers belonging to the Theaceae, Apiaceae, Liliaceae, and Santales families and feed on pollen and nectar. As observed in other hoverflies, species belonging to *Eristalinus* are often involved in entomophily when pollinating and fertilizing plants such as *Eurya emarginata* (Thunb.), *Santalum album* L., *Eryngium horridum* Malme, and *Allium cepa* L.^22–25^. Saprophagy larvae of *Eristalinus* live in various organic-rich substrates, such as around pools, rotting trees, or other plants, and have even colonized a human corpse as shown in a recent study^26^.

Four complete mitogenomes—those of *Eristalinus viridis* (Coquillett, 1998) (GenBank No. MN494096), *E. quinquestriatus* (Fabricius, 1781) (MT471322), *E. tarsalis* (Macquart, 1855) (MW073114), and *E.* sp*.* (MT942687)—were sequenced and described in this study. We analyzed the genomic structure and nucleotide composition of these four sequenced species and compared these with other Syrphidae^14–18,20,27,28^; furthermore, we reconstructed phylogenetic relationships combined with current mitochondrial genomes. This study aims to compare and elucidate the phylogenetic relationships among *Eristalinus* and Syrphidae.

## Materials and methods

### Ethics statement

The specimens studied here were collected in the field by net. The field work permission for specimen collection to *Eristalinus viridis*, *E. quinquestriatus*, *E. tarsalis* was approved by the Changqing National Nature Reserve, Hanzhong, Shaanxi, China, and was performed in accordance with relevant guidelines of the reserve, that for *Eristalinus* sp. is needless due to the location was not privately-owned or protected.

### Sampling, genomic DNA extraction, and polymerase chain reaction (PCR) amplification

Voucher specimens were deposited in the Museum of Zoology and Botany, Shaanxi University of Technology, Hanzhong, China. Specimens of *E. viridis*, *E*. *quinquestriatus*, *E*. *tarsalis,* and *E.* sp. were collected from Shaanxi Province, China (Table S1), identified by Hu Li and Juan Li using the works by Huo et al*.*^2^ and Huang and Cheng^3^. After collection, specimens were transported to the laboratory in absolute ethanol and stored at − 20 °C.

Genomic DNA was extracted from adult’s thorax and legs using the TIANamp Genomic DNA Kit (TIANGEN, Beijing), and the sample volume was 100 µl for each species. Specific experimental procedures were strictly carried out following the manufacturer’s instructions. Genomic DNA concentration reached 20 ng/µl or more, then at least 50 µl of the sample was sent to Berry Genomics (Beijing, China) for sequencing, the entire mitogenomes of the four species were sequenced using an Illumina NovaSeq6000 platform with 150 bp paired-end reads and insert size of 350 bp, and all four voucher specimens generated 6 GB high-throughput data.

The remaining sample was used for PCR amplification of *COI* by Sangon Biotech (Shanghai, China). The *COI* sequence as a bait sequence was used to obtain the whole mitogenomes sequence. Taq PCR Master Mix (2 × , blue dye) (BBI Life Sciences, Shanghai) was used in the PCR amplification of *COI*. The primers used for PCR amplification of the *COI* gene were universal for invertebrate phyla (Table S2)^29^. PCR amplification included an initial denaturing step at 94 °C for 4 min, followed by 35 cycles of denaturation at 94 °C for 30 s, annealing at 45 °C for 30 s, elongation at 72 °C for 45 s, and a final elongation step at 72 °C for 10 min. PCR amplification procedures of *COI* were carried out following the manufacturer’s manual.

### Mitochondrial genome sequencing, assembly, and annotation

Complete mitogenomes were assembled using Geneious Prime (v2019 1.3.)^30^ combined with PCR amplification of *COI* sequences while using *Eristalinus aenax* (MH321208) and *Eristalinus tabanoides* (MH321207) as references to confirm the accuracy.

The secondary structure and position of 22 tRNAs were predicted by ARWEN version 1.2^31^ and tRNAscan-SE version 1.21^32^ and were checked manually. Those tRNAs that could not be found were confirmed by alignment with homologous sequences from related species. PCGs were annotated with Geneious Prime (v2019 1.3.)^30^ by detecting an open reading frame, which was also confirmed based on BLAST query in GenBank using a published mitogenome from Syrphidae. For rRNA gene identification, the *16S* rRNA gene was located between the *tRNA*-*L2* and *tRNA*-*V*; the *12S* rRNA gene was identified based on comparison with other related species. The secondary structures of *16S rRNA* and *12S rRNA* were predicted according to data from other species, tobacco hornworm, *Apis mellifera*, *Scopura longa*, and *Andrena chekiangensis*^33–36^. Helical elements were predicted using ClustalX 1.81^37^ and RNA Structure 5.2^38^. The control region was identified by the boundaries of *tRNA-I* and *12S* rRNA.

The four species’ mitogenome maps in this study were produced using CG View online server using default parameters (http://stothard.afns.ualberta.ca/cgview_server/)^39^. Nucleotide composition was calculated using MEGA 6.0^40^. The AT and GC skew were calculated manually according to formulas: AT skew = (A% − T%)/(A% + T%) and GC skew = (G% − C%)/(G% + C%)^41^. The codon usage and relative synonymous codon usage (RSCU) of each PCGs were calculated using MEGA 6.0^40^. Homology between control region repeat units in *Eristalinus* species with the control region of other species was determined using a ClustalW^37^ sequence alignment implemented in MEGA 6.0^40^. The numbers of nonsynonymous substitutions per nonsynonymous site (Ka) and synonymous substitutions per synonymous site (Ks) were calculated for Syrphidae species using DnaSP v4^42^. The ratio of Ka/Ks was checked manually.

### Phylogenetic analysis

A total of 24 species mitogenomes of Syrphidae, including four newly sequenced *Eristalius* species, were used for phylogenetic analyses (Table 1). *Nemopoda mamaevi* (Sepsidae)^43^ and *Cestrotus liui* (Lauxaniidae)^44^ were used as outgroups.

**Table 1:** Information of complete mitogenomes used for phylogenetic analysis in this study.

	Species	Accession number	Length/bp	Reference
Syrphidae	*Episyrphus balteatus*	KU351241	16,175	Pu et al.^20^
	*Eristalis cerealis*	NC050932	15,348	Yan et al.^27^
	*Eristalis tenax*	MH159199	15,996	Li et al.^21^
	*Eristalinus barclayi*	MH321205	15,757	Sonet et al.^14^
	*Eristalinus fuscicornis*	MH321204	15,815	Sonet et al.^14^
	*Eristalinus vicarians*	MH321206	15,966	Sonet et al.^14^
	*Eristalinus aeneus*	MH321208	16,245	Sonet et al.^14^
	*Eristalinus tabanoides*	MH321207	15,792	Sonet et al.^14^
	*Eristalinus quinquestriatus*	MT471322	15,872	This study
	*Eristalinus viridis*	MN494096	15,640	This study
	*Eristalinus* sp.	MT942687	15,883	This study
	*Eristalinus tarsalis*	NW073114	15,849	This study
	*Eristalinus quinquestriatus*	MT834869	16,198	Unpublished
	*Eupeodes corolla*	KU379658	15,326	Pu et al.^20^
	*Helophilus virgatus*	MN148445	15,742	Li et al.^22^
	*Korinchia angustiabdomena*	MK870078	16,473	Li ^23^
	*Melanostoma orientale*	MN788095	16,229	Chen et al.^24^
	*Melanostoma scalare*	MN481591	16,126	Unpublished
	*Ocyptamus sativus*	KT272862	15,214	Junqueira et al.^25^
	*Phytomia zonata*	MT478107	15,716	Li et al.^26^
	*Platycheirus albimanus*	MT622646	16,648	Unpublished
	*Simosyrphus grandicornis*	DQ866050	16,141	Cameron et al.^10^
	*Ferdinandea cuprea*	MT834868	15,907	Unpublished
	*Volucella nigricans*	MK870079	15,724	Li ^23^
Outgroup	*Nemopoda mamaevi*	NC026866	15,878	Li et al.^42^
	*Cestrotus liui*	NC034922	16,171	Li et al.^43^

Each PCG was aligned individually with codon-based multiple alignments using the MAFFT algorithm in the TranslatorX server^45^ (http://pc16141.mncn.csic.es/index_v4.html). The two rRNA genes were aligned using the MAFFT v7 online server with G-INS-i strategy^46^, and poorly aligned positions were eliminated using Gblocks 0.91b^47^ (https://mafft.cbrc.jp/alignment/server/). Finally, the aligned sequences of 13 PCGs and two rRNA genes were concatenated manually with MEGA 6.0^40^.

PartitionFinder2^48^ on CIPRES Science Gateway^49^ was used to select the best-fit partitioning schemes and substitution models for the dataset matrix. The greedy algorithm was employed using unlinked branch lengths, and under the Akaike information criterion to select the optimal partitioning model. Information concerning partition strategies and substitution models used are summarized in Table S3. Phylogenetic trees were constructed using the maximum likelihood method (ML) on the IQ-TREE webserver (http://www.iqtree.cibiv.univie.ac.at/) ^50^and Bayesian inference (BI) on MrBayes 3.2.6^51^ within the CIPRES webserver (https://www.phylo.org/portal2/login!input.action) ^49^based on the sequences of 13 PCGs and two rRNA genes (PCGRNA, PCG123, PCG12, PCG12RNA, and AA datasets), respectively.

PCGRNA indicates the sequences from PCGs and rRNAs; PCG123 represents all bases PCGs: PCG12 represents all bases excluding the third bases of each protein-coding amino acid genes; PCG12RNA represents the sequences of PCG12 and rRNA. AA represents the amino acids translated from 13 PCGs.

The ML analyses were conducted by using 10,000 replications with the “ultrafast” function^52^. For BI analyses, two simultaneous Markov chain Monte Carlo runs running parameters ran 1 million generations with sampling every 1,000 generations. The initial 25% of the sampled data were discarded as burn-ins.

## Results and discussion

### Genome organization and base composition

**Figure 1:**
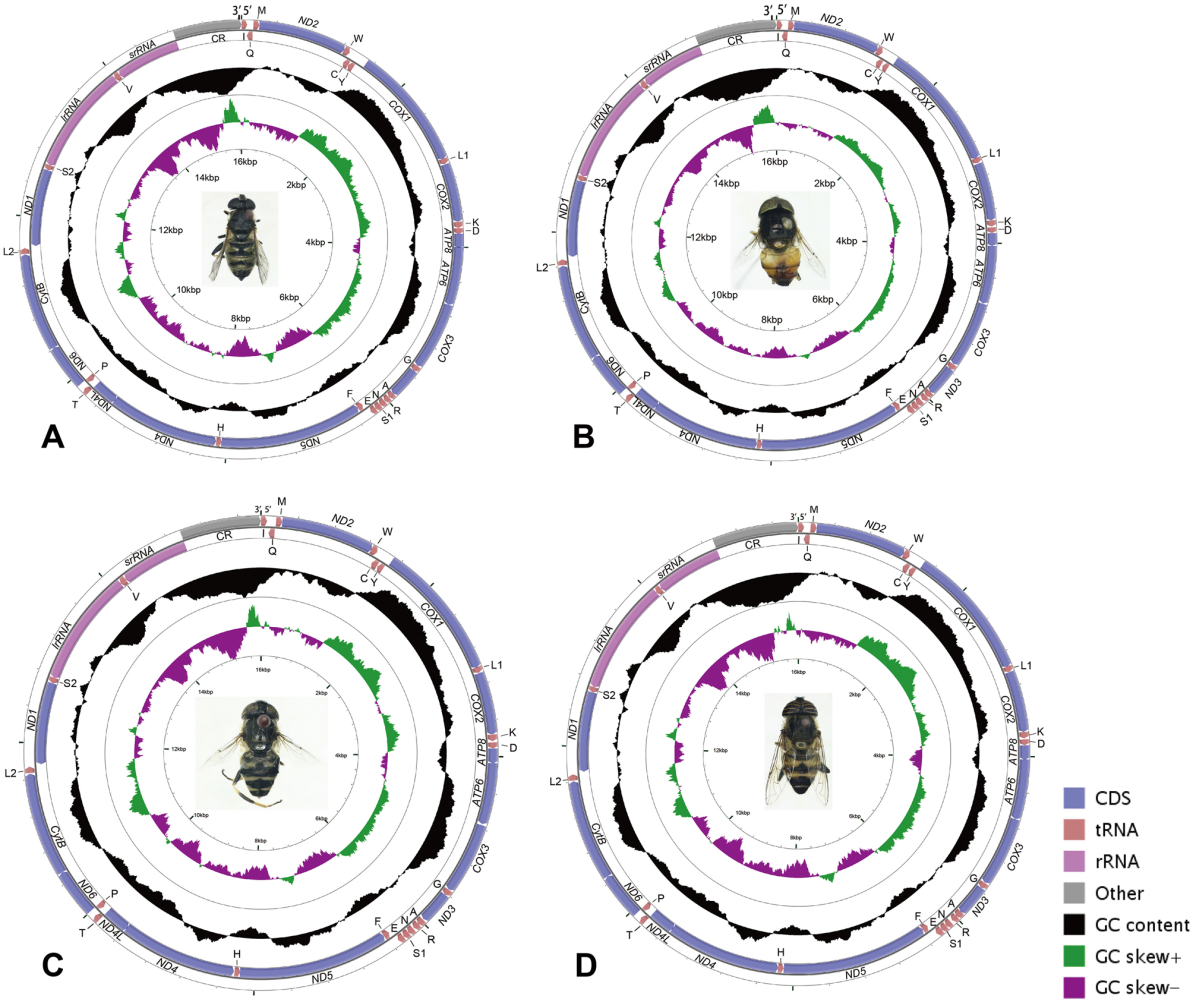
The circle maps of the complete mitochondrial genomes of *Eristalinus*. (**A**) *E. viridis*, 15,640 bp, (**B**) *E. quinquestriatus*, 15,872 bp, (**C**) *E*. *tarsalis*, 15,849 bp, (**D**) *E.* sp., 15,883 bp. PCGs, tRNAs, rRNAs have been given, CR represented control region. The arrow indicates the direction of the gene from start to end. A black pattern shows GC content: four species, pictures taken by Juan Li using CG view (http://stothard.afns.ualberta.ca/cgview_server/).

The complete mitogenomes of *E. viridis*, *E. quinquestriatus*, *E. tarsalis*, and *E.* sp. were sequenced; the total length of each genome was 15,640 bp, 15,872 bp, 15,849 bp, and 15,883 bp respectively, with each mitogenome including 37 genes (13 PCGs, 22 tRNAs, 2 rRNAs) and non-coding regions (Fig. 1, Tables S4, S5, S6, S7). A total of 23 genes were encoded on the J-strand and another 14 were located on the N-strand, which is consistent with the mitogenome sequences of other Syrphidae species^14–18,20,27,28^. Within Diptera, mitogenomes were found to have gene rearrangements in mosquitos (Culicidae)^53,54^ and gall midges (Cecidomyiidae)^23^. The gene arrangement within the mitogenomes of Syrphidae was the same as that within the mitogenome of the ancestral insect (*Drosophila yakuba*)^55^. The length of Syrphidae genomes ranged from 15,326 to 16,473 bp, with *Korinchia angustiabdomena* having the longest genome (16,473 bp) and *Eupeodes corolla* having the shortest (15,326 bp). Overall, the genomic size of the species within these taxa is medium compared with that of other insects.

Within *Eristalinus*, there are 10 complete mitogenomes sequenced in total, including these 4 newly sequenced mitogenomes in the current study and 6 from GenBank. The 10 complete mitogenomes from 9 *Eristalinus* species available have a high A + T content: 80.6% in *E*. *quinquestriatus* (MT834869), 80.2% in *E*. *quinquestriatus* (MT471322, this study), 80.0% in *E*. *tabanoides* and *E*. *vicatians*, 79.9% in *E*. *fusciornis* and *E*. *barclayi*, 79.8% in *E*. *aeneus* and *E.* sp., 78.2% in *E*. *viridis*, and 79.0% in *E. tarsalis*. Furthermore, all species exhibited strong AT bases, and all consisted of positive AT and negative GC skew (Table 2).

**Table 2:** Nucleotide composition of the four sequenced species complete mitogenomes of *Eristalinus*.

Region		Total (bp)	T%	C%	A%	G%	ATskew	GCskew
*E. viridis*	Whole	15,640	37.3	13.1	40.9	8.6	0.046	−0.207
	PCGs	11,122	43.1	11.9	33.0	11.9	−0.133	0
	tRNAs	1481	39.4	8.6	40.5	11.5	0.0134	0.144
	rRNAs	2126	42.9	5.7	39.7	11.7	−0.039	0.345
	Control region	784	43.1	4.7	49.9	2.3	0.073	−0.343
*E. quinquestriatus*	Whole	15,872	39.2	11.6	41.0	8.3	0.022	−0.166
	PCGs	11,168	44.3	10.5	33.9	11.3	−0.133	0.037
	tRNAs	1490	39.7	8.7	39.8	11.8	0.001	0.151
	rRNAs	2130	42.9	5.6	40.8	10.7	−0.025	0.313
	Control region	959	46.4	2.8	48.7	2.1	0.024	−0.143
*E.* sp.	Whole	15,883	38.9	12.0	40.9	8.3	0.025	−0.182
	PCGs	11,170	43.9	10.8	33.8	11.5	−0.12	0.031
	tRNAs	1493	39.4	8.5	40.4	11.7	0.013	0.158
	rRNAs	2139	42.4	5.8	40.6	11.1	−0.022	0.314
	Control region	960	49.6	3.2	45.7	1.5	−0.040	−0.368
*E. tarsalis*	Whole	15,849	38.2	12.6	40.8	8.5	0.033	−0.194
	PCGs	11,167	43.5	11.4	33.2	11.9	−0.134	0.215
	tRNAs	1488	39.3	8.5	40.5	11.8	0.015	0.163
	rRNAs	2126	42.6	5.7	40.4	11.3	−0.027	0.329
	Control region	883	47.3	5.1	45.6	1.9	−0.018	−0.457

### Protein-coding genes and codon usage

Within the four species sequenced, most PCGs began with a typical ATN (T/C/G/A) start codon and ended with a stop codon TAA or incomplete T; the incomplete T is a common stop codon in insects, which modified into complete TAA via posttranscriptional polyadenylation during mRNA maturation^56^ (Tables S4, S5, S6, S7). Within the 10 mitogenome sequences of *Eristalinus* species, *ND1* was relatively conservative and always utilized TTG as the start codon. For stop codons, *ND3* contained TAG, *ND5* ended with an incomplete stop codon T, whereas all others utilized TAA as the stop codon.

**Figure 2:**
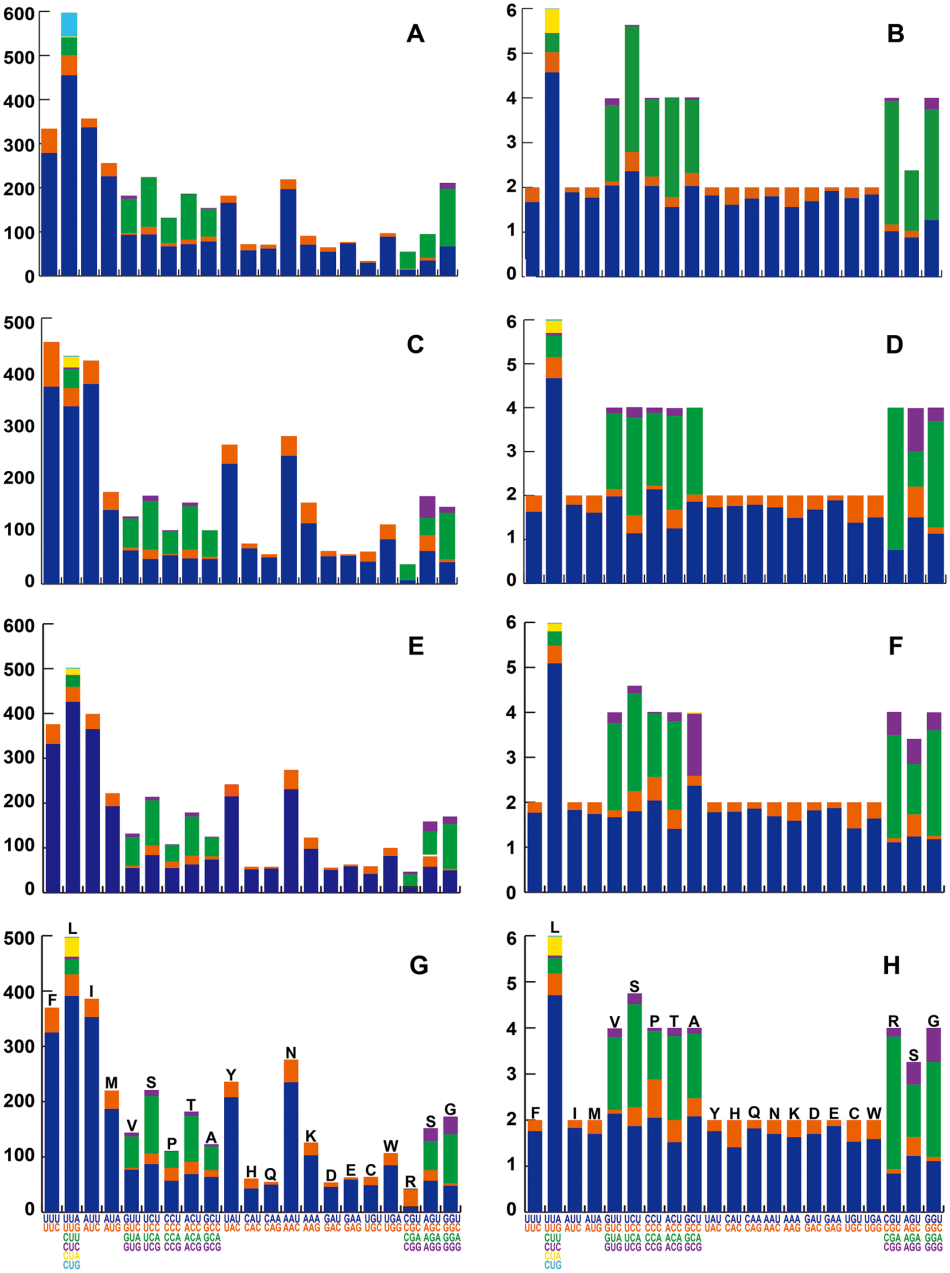
The codon usage (**A**, **C**, **E**, **G**) and Relative Synonymous Codon Usage (RSCU) (**B**, **D**, **F**, **H**) in the mitogenomes of *Eristalinus*. (**A**, **B**) *E. viridis*, (**C**, **D**) *E. quinquestriatus*, (**E**, **F**) *E.* sp., (**G**, **H**) *E*. *tarsalis*. The code color in the horizontal axis corresponds to the same color in the Figures. The image was computed by MEGA 6.0 (http://www.megasoftware.net/previousVersions.php).

PCGs exhibited negative AT (− 0.134 to − 0.12) and positive GC (0–0.037) skew among the four *Eristalinus* species (Table 2). They all exhibited rich A + T content. The most frequently used amino acids were leucine (Leu), phenylalanine (Phe), and isoleucine (Ile) within the four sequenced mitogenomes of *Eristalinus* (Fig. 2). Summarization of the RSCU showed that each gene codon usage exhibited a strong AT bias and was primarily composed of the nucleotides A and T. The most frequently used codons for all amino acids utilized the nucleotides A or T in the third codon positions (Fig. 2).

**Figure 3:**
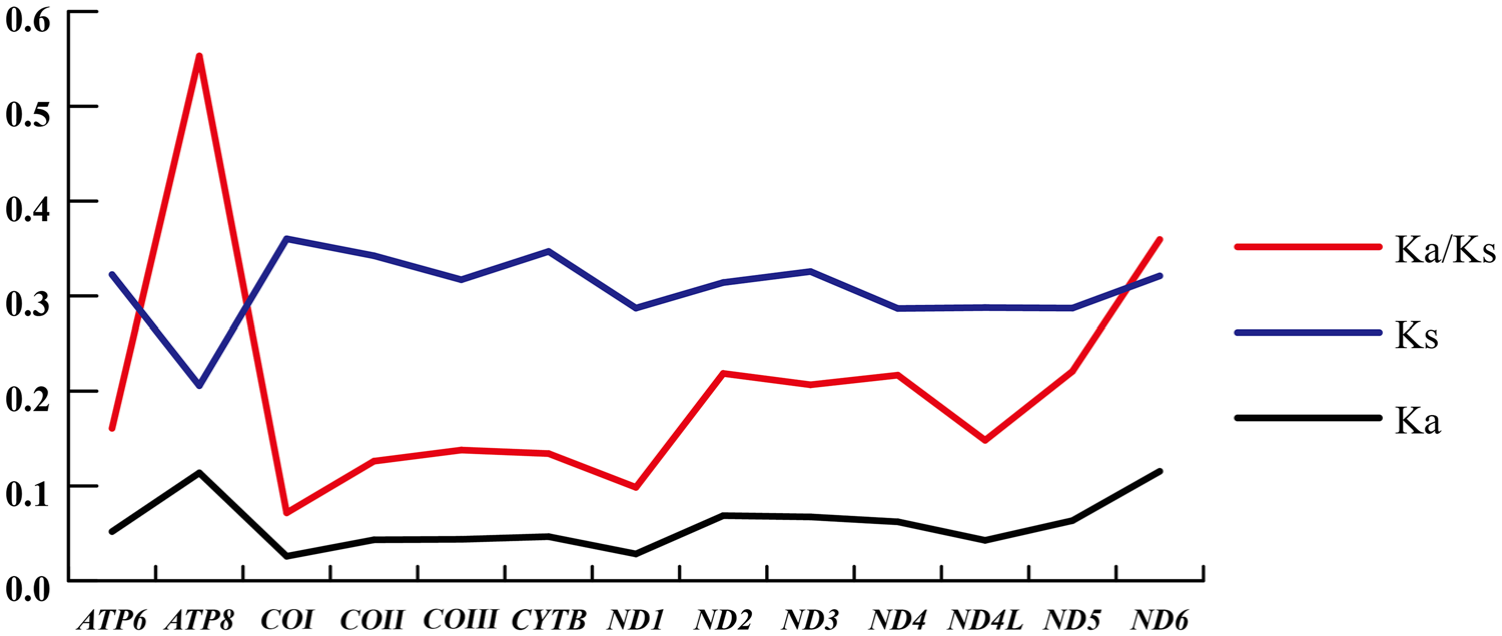
The Ka/Ks analysis of 13 protein-coding genes of the mitochondrial genomes of Syrphidae. The image was computed by DnaSP v4 (http://www.ub.es/dnasp).

Ka/Ks (ω) analysis is a statistical diagnostic method used to detect the form of sequence evolution^57,58^. The 13 PCGs in the Syrphidae mitochondrial genome have values of Ka/Ks < 1, indicating that all these PCGs are under purifying selection (Fig. 3). The gene *ATP8* (ω = 0.553) was predicted to have evolved most rapidly, followed by *ND6* (ω = 0.360), *ND5* (ω = 0.221), *ND2* (ω = 0.219), and *ND4* (ω = 0.217); the gene *COI* (ω = 0.0712) was shown to be the most conservative. Concerning gene-specific substitution rates, Ks ranged from 0.206 at gene *ATP8* to 0.360 at gene *COI*, while the Ka varied from 0.023 at gene *COI* to 0.115 at gene *ND6*. Because the selection pressures upon *ATP8* and *ND6* are relatively weak, and these genes accordingly are relatively unconserved, *COI* and *ND1* are under strong selection pressures and are therefore more conservative, consistent with other Diptera species^59^.

### tRNAs and rRNAs

**Figure 4:**
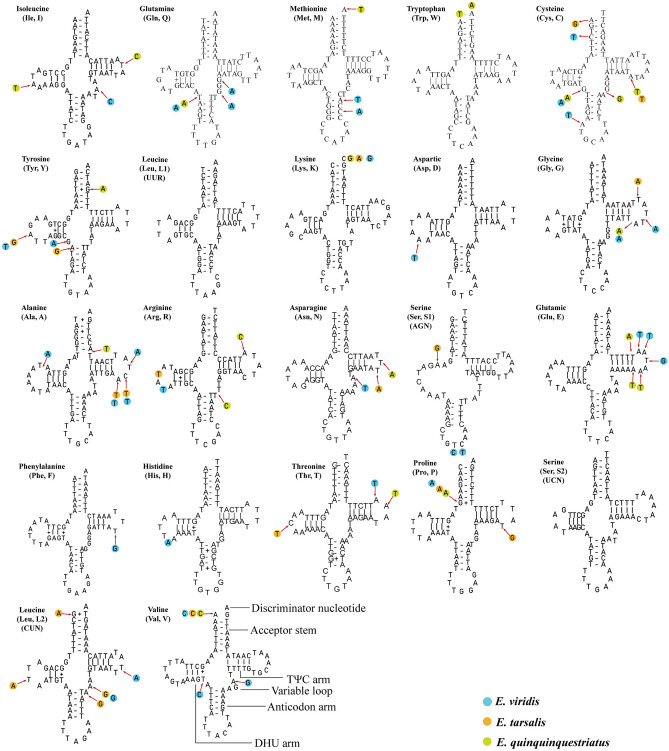
Predicted secondary cloverleaf structure for tRNA of four *Eristalinus* species. (**A**) Isoleucine, (**B**) Glutamine, (**C**) Methionine, (**D**) Tryptophan, (**E**) Cysteine, (**F**) Tyrosine, (**G**, **U**) Leucine, (**H**) Lysine, (**I**) Aspartic, (**J**) Glycine, (**K**) Alanine, (**L**) Arginine, (**M**) Asparagine, (**N**, **T**) Serine, (**O**) Glutamic, (**P**) Phenylalanine, (**Q**) Histidine, (**R**) Threonine, (**S**) Proline, (**V**) Valine. Arrows indicate variations of each site in four species of *Eristalinus*. Each species is marked by unique color (see color legend). The image was predicted by ARWEN version 1.2 (http://130.235.244.92/bcgi/arwen.cgi) and tRNAscan-SE version 1.21 (http://lowelab.ucsc.edu/tRNAscan-SE), drawing with Adobe Illustrator 2020.

Among 22 tRNAs, 8 were encoded on the N-strand and the remaining were encoded on the J-strand (Fig. 1; Tables S4, S5, S6, S7). When comparing the tRNA genes of Syrphidae, the length of all tRNAs was found in the range of 58–72 bp (*tRNA*-*Lys* in *Ocyptamus sativus* and *tRNA*-*Val* in 24 sequenced Syrphidae species, respectively). The secondary structure of tRNA genes was a typical cloverleaf structure including a discriminator nucleotide, acceptor stem, TψC arm, variable loop, anticodon arm and DHU arm (Fig. 4). In the four sequenced species, the DHU arm was found to be missing in only the *tRNA*-*S1* gene, whereas the remaining were standard structures, consistent with those of other Syrphidae^14,16,17,27^ (Fig. 4). In addition, base mismatches were found in all four species sequenced. 12 G–T and 6 T − T mismatches were found in *E. viridis*, as well as 18 G–T and 5 T − T mismatches found in *E. quinquestriatus*, 18 G–T, 3 T–T, 1 A–G, and 1 C–T mismatches found in *E.* sp., 17 G–T and 4 T − T mismatches found in *E*. *tarsalis* (Fig. 4).

The nucleotide composition of these 22 tRNA genes was significantly biased to A and T nucleotides. The *E. viridis* mitogenome contains 1,481 bp with an A + T content of 79.9%, the *E. quinquestriatus* contains 1,490 bp with an A + T content of 79.5%, the *E.* sp. mitogenome contains 1,493 bp with an A + T content of 79.8%, the *E*. *tarsalis* mitogenome contains 1,488 bp with an A + T content of 79.8% (Table 2). Four species exhibit positive AT and GC skew.

The two ribosomal RNA genes contain both *16S* and *12S* rRNA, between either the *tRNA*-*L2* and *tRNA*-*V* or between the *tRNA*-*V* and control region, respectively (Fig. 1). Within the Syrphidae mitogenome, the *16S* rRNA length ranged from 1,313 bp (*Ornithopus sativus*) to 1,414 bp (*Melanostoma scalare*), and that of *12S* rRNA ranged from 778 bp (*O. sativus* and *M. scalare*) to 824 bp (*K. angustiabdomena*).

For *E. viridis*, the length of *12S* and *16S* rRNA was 793 bp and 1,333 bp, respectively, with an A + T content of 82.6%. Within *E. quinquestriatus*, the *12S* and *16S* rRNA were 793 bp and 1337 bp long, with an A + T content of 83.7%; in *E.* sp., the *12S* and *16S* rRNA were 793 bp and 1,346 bp in length, with an A + T content of 83.0%; and in *E*. *tarsalis*, the *12S* and *16S* rRNA were 790 bp and 1,336 bp long, with an A + T content of 83.0% (Table 2).

**Figure 5:**
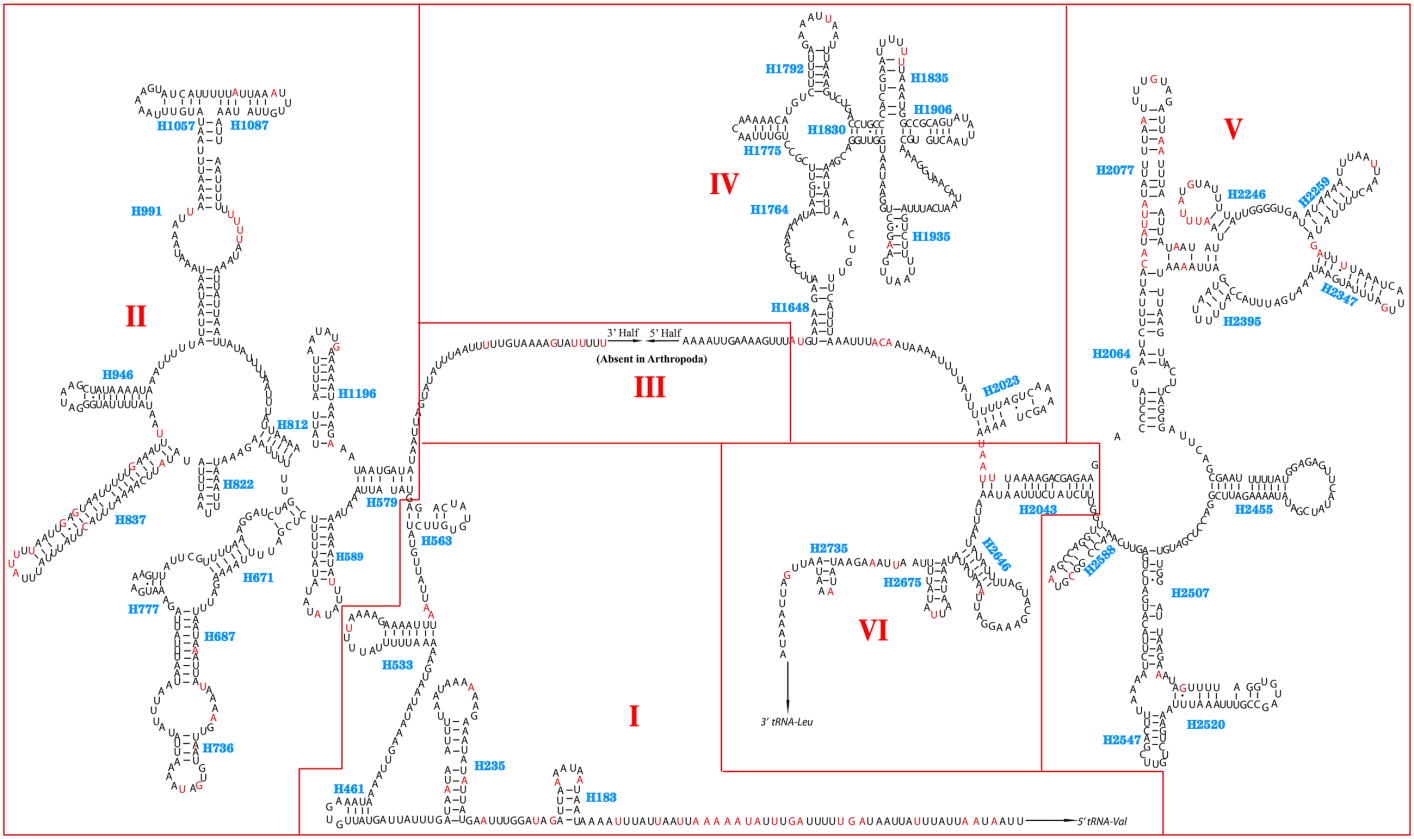
Predicted secondary structure for *16S* rRNA of *E.* sp. The red color indicates the variation of nucleotide sites in four species. The names of helices are shown in blue “H + numbers.” Areas surrounded by red lines indicate different domains and are respectively numbered I, II, IV, V, and VI in red as in other insects. The image was predicted by ClustalX 1.81 (http://www.hgmp.mrc.ac.uk/Registered/Option/clustalx.html) and RNA Structure 5.2 (http://rna.urmc.rochester.edu/RNAstructure.html), drawing with Adobe Illustrator 2020.

Among the four species of *Eristalinus*, the secondary structure of *16S* rRNA includes 5 domains (I, II, IV, V, and VI; domain III was absent in arthropods.) and 43 helices (Fig. 5). Multiple alignments of four species’ *16S* rRNA gene extended over 1,350 positions and included 1,242 conserved and 108 variable sites. Domain IV was more conserved than other domains structurally.

**Figure 6:**
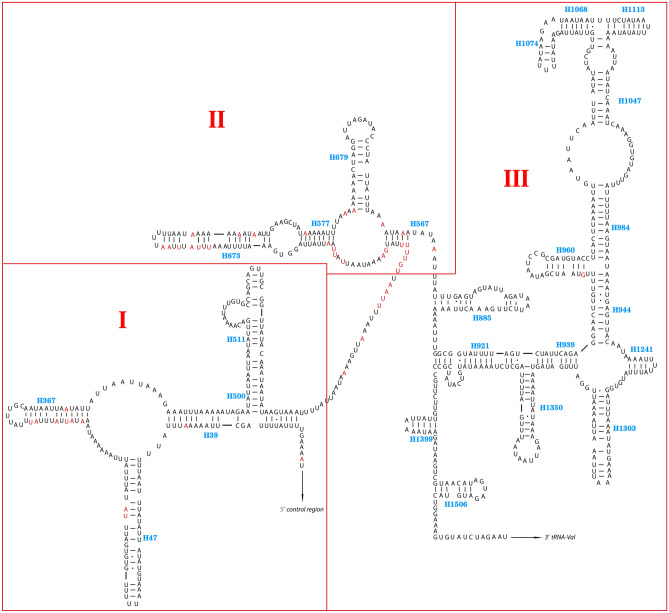
Predicted secondary structure for *12S* rRNA of *E.* sp. The red color indicates the variation nucleotide sites in four species. The names of helices showed in blue “H + numbers.” Areas surrounded by red lines indicate different domains and are respectively numbered I, II, and III in red as in other insects. The image was predicted by ClustalX 1.81 (http://www.hgmp.mrc.ac.uk/Registered/Option/clustalx.html) and RNA Structure 5.2 (http://rna.urmc.rochester.edu/RNAstructure.html), drawing with Adobe Illustrator 2020.

The secondary structure of *12S* rRNA contains 3 domains (I, II, and III) and 24 helices (Fig. 6). Multiple alignments of the four species’ *12S* rRNA gene extended over 700 positions and comprised 655 conserved and 45 variable sites. Domain III was more conserved than other domains structurally.

### Non-coding region

The non-coding region contains two parts: gene intervals and a control region (CR; AT-rich region). The *E. viridis* mitogenome contains 10 gene intervals ranging from 1 to 13 bp and has 15 pairs of gene overlaps ranging from 1 to 29 bp (Table S4). The mitogenome of *E. quinquestriatus* contains 12 gene intervals ranging from 1 to 9 bp, with 15 pairs of gene overlaps ranging from 1 to 32 bp (Table S5). The *E.* sp. mitogenome contains 14 gene intervals ranging from 1 to 34 bp and has 11 pairs of gene overlaps ranging from 1 to 9 bp (Table S6). The *E. tarsalis* mitogenome contains 19 gene intervals ranging from 1 to 36 bp and has 7 pairs of gene overlaps ranging from 1 to 7 bp (Table S7).

The CR is the largest non-coding region with the largest variation in the entire mitochondrial genome sequence and length. It is primarily involved in mitochondrial DNA replication and transcription^60^. The CRs of *E. viridis*, *E. quinquestriatus*, *E.* sp., and *E. tarsalis* are 784 bp, 959 bp, 960 bp, and 833 bp in length, respectively (Table 2). This region was shown to be significantly rich in AT with an A + T content of 93.0% in *E. viridis*, 95.1% in *E. quinquestriatus*, 95.3% in *E.* sp*.*, 92.9% in *E*. *tarsalis* (Table 2). The CRs of the four species showed slightly positive AT skews and negative GC skews. The CR length in the *Eristalinus* genus is highly variable, ranging in size from 735 bp (*E*. *tabanoides*) to 1284 bp (*E. quinquestriatus* (MT834869)).

**Figure 7:**
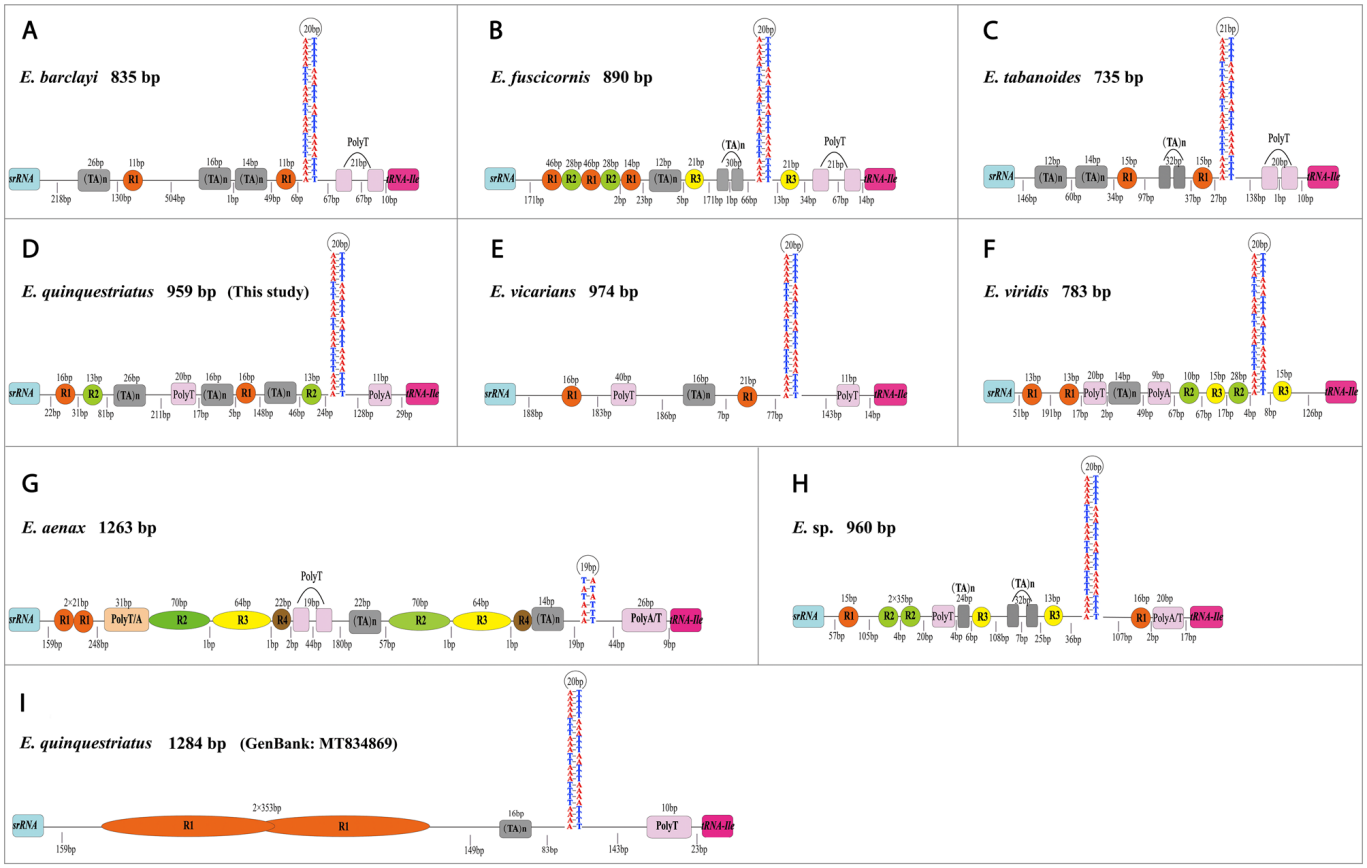
Control Regions (CRs) of *Eriatalinus*. (**A**) *E. barclayi*, (**B**) *E. fuscicornis*, (**C**) *E. tabanoides*, (**D**) *E. quinquestriatus*, (**E**) *E*. *vicarians*, (**F**) *E. viridis*, (**G**) *E. aenax*, (**H**) *E.* sp., (**I**) *E. quinquestriatus*. Different shapes and colors represent the different kinds of sequences. “R” refers to repeat units. The image was predicted by Geneious Prime 2019 (www.geneious.com), drawing with Adobe Illustrator 2020.

In this study, the CRs of the 10 complete mitogenomes were also observed to contain some structural elements (Fig. 7), such as poly-T/A stretch, stem-loops, and tandem repeats^60^. Nearly all species contained poly-T/A structures in random positions—except for *E*. *viridis*—with at least one poly-T/A structure near the *tRNA*-*I*. Stem-loop structures were found close to the boundary of the *tRNA*-*Ile* (Fig. 7). When comparing CRs of the *Eristalinus* species, we found a conservative sequence of 68 bp containing a stem-loop structure (Fig. S7). Within the CRs of 10 mitogenomes, the “(TA)_n_” (n ≥ 5) motif was common (Fig. 7). There were multiple repetitive units observed, and while only *E*. *aenax* (Fig. 7G) and *E. quinquestriatus* (GenBank: MT834869) (Fig. 7I) contained one tandem repeat unit, those repeat units within the other species were scattered throughout the CRs (Fig. 7). The number of repeat units varied (*E*. *tarsalis* has not repeat units), *E*. *aenax* contained the largest number with four repeat units (Fig. 7G). *E*. *fuscicornis* (Fig. 7B), *E*. *quinquestriatus* (Fig. 7D), *E. viridis* (Fig. 7F), and *E.* sp. (Fig. 7H) contained the least with one repeat unit. The repeat unit genomic locations for each species were random. The longest repeat unit sequence was 353 bp long and the shortest was 11 bp long (Fig. 7).

### Phylogenetic analysis

**Figure 8:**
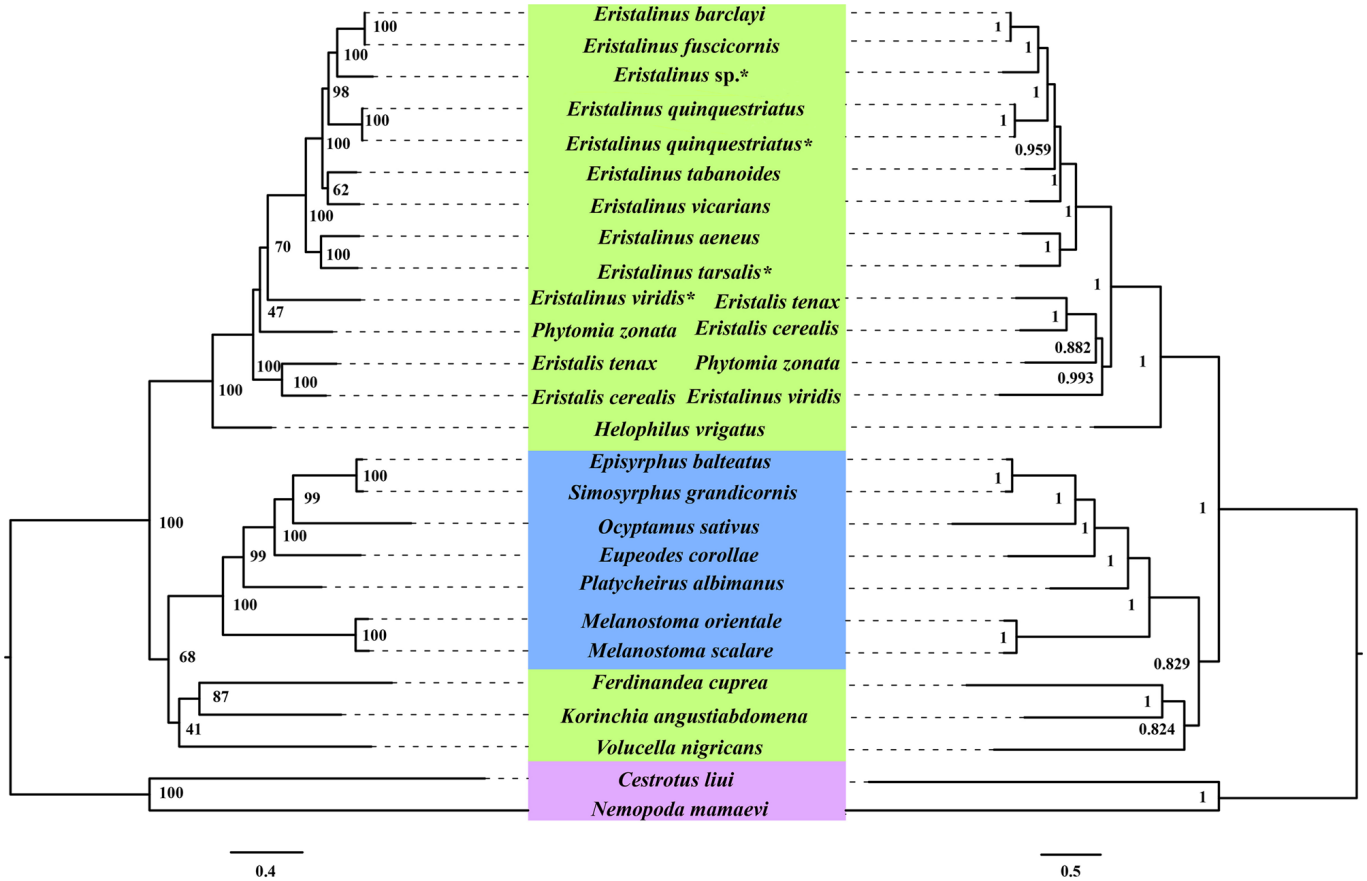
Phylogenetic trees of Syrphidae based on the PCGRNA dataset using maximum likelihood (ML, left) and Bayesian inference (BI, right) methods. “Bootstrap support” (ML) and “posterior probabilities” (BI) are indicated at their nodes respectively. The species sequenced in this study were marked with * after the species name. The image was mainly predicted by IQ-TREE (http://iqtree.cibiv.univie.ac.at/) and MrBayes in CIPRES webserver (https://www.phylo.org/portal2/login!input.action), drawing with Adobe Illustrator 2020.

Phylogenetic analysis employing the PCGRNA (13,187 bp), PCG123 (11,106 bp), PCG12 (7,404 bp), PCG12RNA (9,485 bp), and AA (3701 amino acids) datasets from 24 hoverflies and 2 outgroup species showed nearly similar topologies with strong node support under both ML and BI methods (Figs. 8, S8, S9, S10, S11). Because the topology of the PCGRNA datasets significantly conforms to the morphological classification and more previous research, trees from the PCGRNA dataset were chosen in our study^14–19^. In this study, we provided more mitogenome sequences and datasets conducive to understanding the relationship within Syrphidae.

The subfamily Syrphinae has been reconstructed into a monophyletic group supported in both ML and BI analyses [bootstrap support values (BS) = 68, Bayesian posterior probability (pp = 0.829)] (Fig. 8). The tribes Syrphini and Melanostomini are sister taxa with strong support and the six genera relationship of Syrphinae—(*Melanostoma* + (*Platycheirus* + (*Eupeodes* + (*Ocyptamus* + (*Simosyrphus* + *Episyrphus*)))—are strongly supported, and these results are consistent with the previous studies^14,20–23,26^.

Two main types of clades can be observed in all phylogenetic trees except BI analyses based on the AA dataset (Figs. 8, S8, S9, S10, S11). One included the tribe Volucellini that diverged first, then Milesiini and Cheilosiini, and their phylogenetic relationships are as follows: (Syrphinae + (*Volucella* + (*Ferdinandea* + *Korinchia*))) (Figs. 8, S8, S9, S10, S11). Furthermore, the other one was that 14 species of Eristalini were clustered as a single clade with a higher internal node (Figs. 8, S8, S9, S10, S11). *Volucella nigrican* was the sister species to the other Syrphidae only in BI analyses based on the AA dataset (Fig. S11). All Eristalinae species were not gathered into one clade in all 10 trees based on different datasets of mitogenome sequences datasets. According to the topologies obtained from five datasets based on ML and BI methods, Eristalinae tends to be a paraphyletic group. A phylogenetic relationship study of 27 species of Diptera based on *16S*, *12S*, and morphology^8^ showed Eristalinae to be paraphyletic. Based on *COI*, *28S*, *18S*, and 111 adults morphological, the phylogenetic relationships indicate that Eristalinae was resolved as non-monophyletic^6^. Eristalinae larvae feeding habits are complex; mitogenomes in the phylogenetic relationship in subfamily Eristalinae contain three tribes (Eristalini, Cheilosiini, and Volucellini). Eristalini larvae are aquatic saprophages^2^, Volucellini larvae specialized inquilines in social insect nests, and some feed on dead or dying insects^3,61^. The feeding habits of Volucellini larvae may be close to those of Syrphinae.

Within Eristalini, phylogenetic trees contained four genera in this study, its major phylogenetic relationships congruently cluster as (*Helophilus* + (*Eristalis* + (*Phytomia* + *Eristalinus*))) (Fig. 8, S8, S9, S10, S11); this result was consistent with the relationships based on *COI* by Sonet et al.^14^, but disagreed with that based on *Cyt b* by Zhang & Huo^21^, in which, *Phytomia* diverged first, and *Eristalis* and *Eristalinus* clustered as a sister group, with the cluster formed as (*Phytomia* + (*Helophilus* + (*Eristalis* + *Eristalinus*))). In this study, *Helophilus* is a sister clade to other genera within the Eristalini group and was highly supported [BS = 100, pp = 1] (Fig. 8, S8, S9, S10, S11).

In the genus *Eristalinus*, the seven topologies generated from all five datasets have shown that *E*. *viridis* is the sister species of other species (Fig. 8, S8, S9, S10, S11). Furthermore, *E. fuscicornis*, *E*. *barclayi*, *E*. *tarsalis* and *E*. *aeneus* are sister species with strong bootstrap support values and posterior probabilities in pair (Fig. 8, S8, S9, S10, S11), it is almost identical with *Sonet *et al.^14^.

The relationships within the genus *Eristalinus* from 3 ML (PCG12RNA, PCG123, and PCG12 datasets) and 2 BI (PCG12 and PCG12RNA datasets) phylogenetic trees are inconsistent, *E. tabanoides* and *E*. *vicarians* are gathered in a sister relationship, but it is not exposed in other five trees. *E. viridis*’ status may need to be further verified and discussed because it gathered into the clade under the genus *Eristalinus* in most seven trees but not in only three trees (AA, PCGRNA and PCG123 datasets from BI inference). These issues may be related to the selections of datasets, the different methods (ML and BI), or limited numbers of complete mitogenomes in the family Syrphidae^62,63^. The morphological characteristic of *E. viridis* is differences with other *Eristalinus*, which its eyes without any spots. This characteristic may suggest its unstable branch in phylogenetic relationship.

## Conclusions

The complete mitogenomes of *E. viridis*, *E. quinquestriatus*, *E.* sp., and *E*. *tarsalis* were sequenced and described in the present study. No gene arrangement was found in either of these sequences, and the gene order and direction were similar to the arthropod ancestral mitochondrial genome. Among *Eristalinus*, a conserved stem-loop structure exists near the 3′ end of the CR.

Phylogenetic analyses of 24 Syrphidae species support the monophyly of Syrphinae but do not support that of Eristalinae based on ML and BI methods using five datasets. The phylogenetic relationships constructed using the complete mitogenomes effectively interpreted the genus-level relationships within Eristalini, stating that species of *Helophilus* diverged first, followed by *Eristalis*, *Phytomia* and *Eristalinus*. The complete mitochondrial genomes sequenced in this study provided valuable data, which would be useful for determining the phylogenetic relationship of Syrphidae in the future. Thus, additional mitochondrial genome sampling and more molecular data are still required in order to effectively resolve the phylogeny and lineages of Syrphidae species.

## Supplementary Information

Supplementary Information 1.

Supplementary Information 2.

## Data Availability

The complete mitogenomes of *E. viridis*, *E. quinquestriatus*, *E. tarsalis*, and *E.* sp. were deposited in the GenBank database under accession numbers MN494096, MT471322, MW073114, and MT942687 respectively.
