# Supplementary material for: Comparative analysis of four complete mitogenomes from hoverfly genus Eristalinus with phylogenetic implications
Source: Sci Rep. 2022 Mar 9;12:4164. doi: 10.1038/s41598-022-08172-6 (PMC8907203; doi:10.1038/s41598-022-08172-6)
Supplement: Supplementary file 1 — Supplementary Information 1. [file 41598_2022_8172_MOESM1_ESM.docx]

**
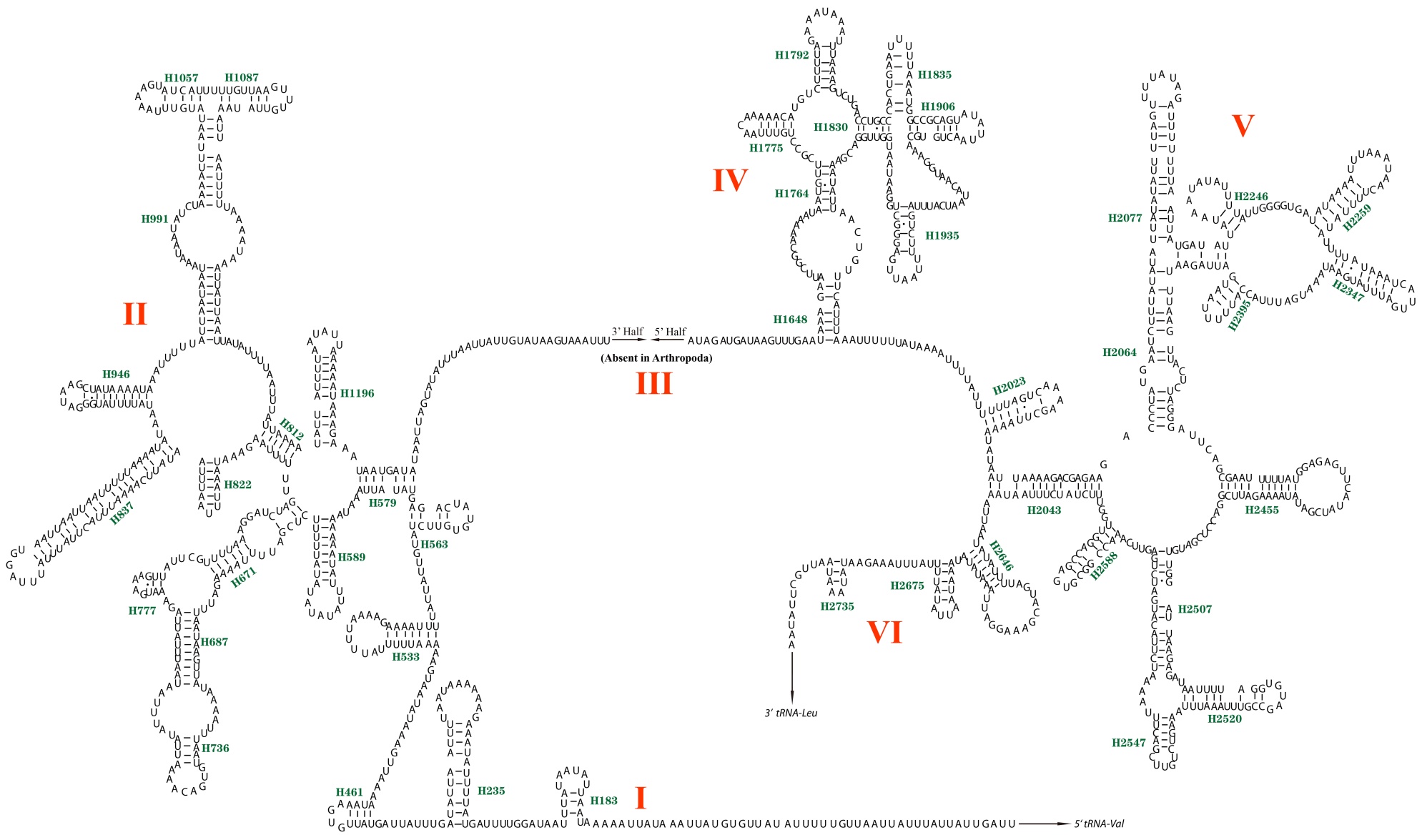
**

**Figure S1.** Predicted secondary structure for *16S* rRNA of *E*. *viridis*. The names of helices are shown in green “H+numbers.”


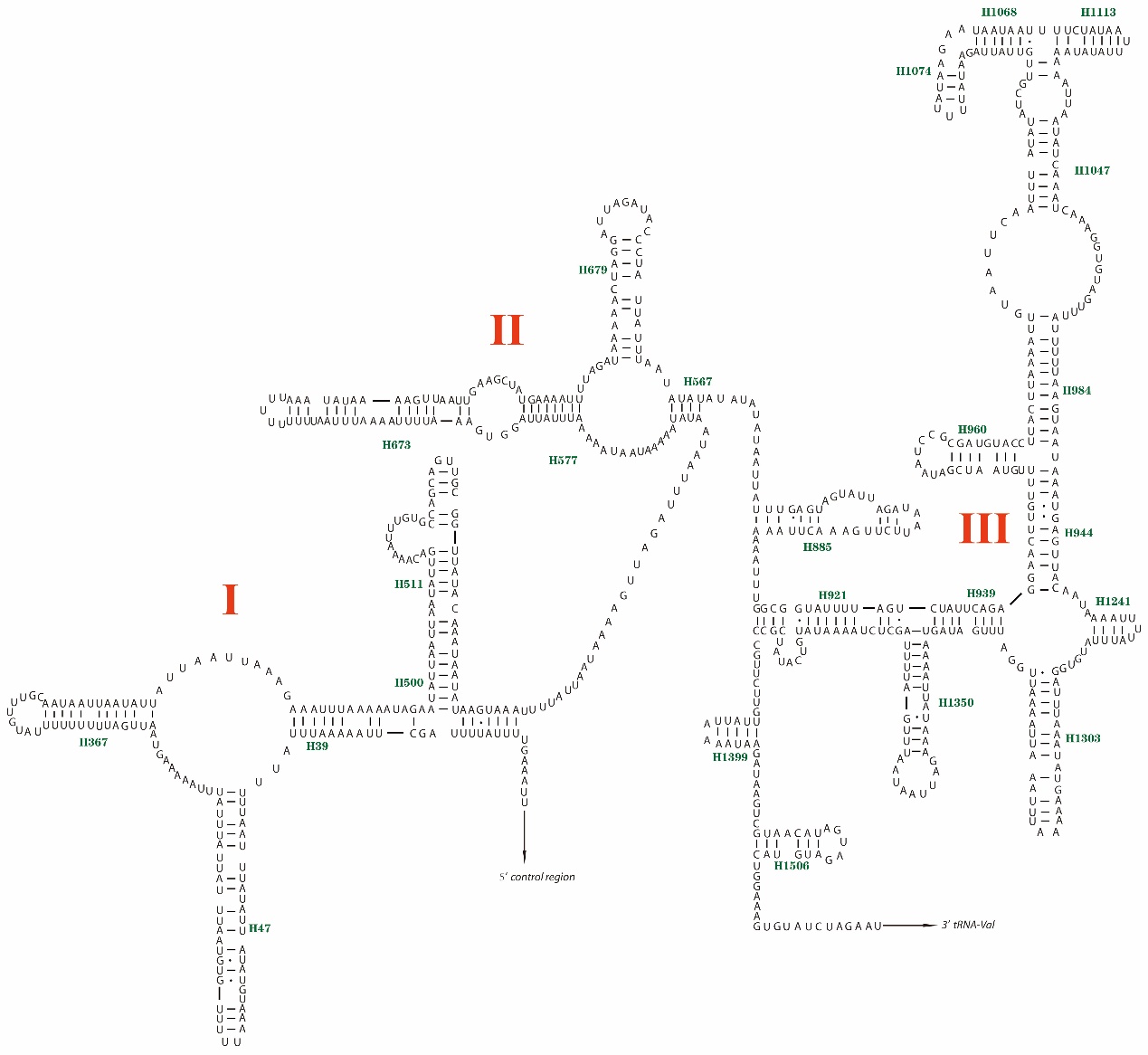


**Figure S2.** Predicted secondary structure for *12S* rRNA of *E. viridis*. The names of helices green shown in green “H+numbers.”


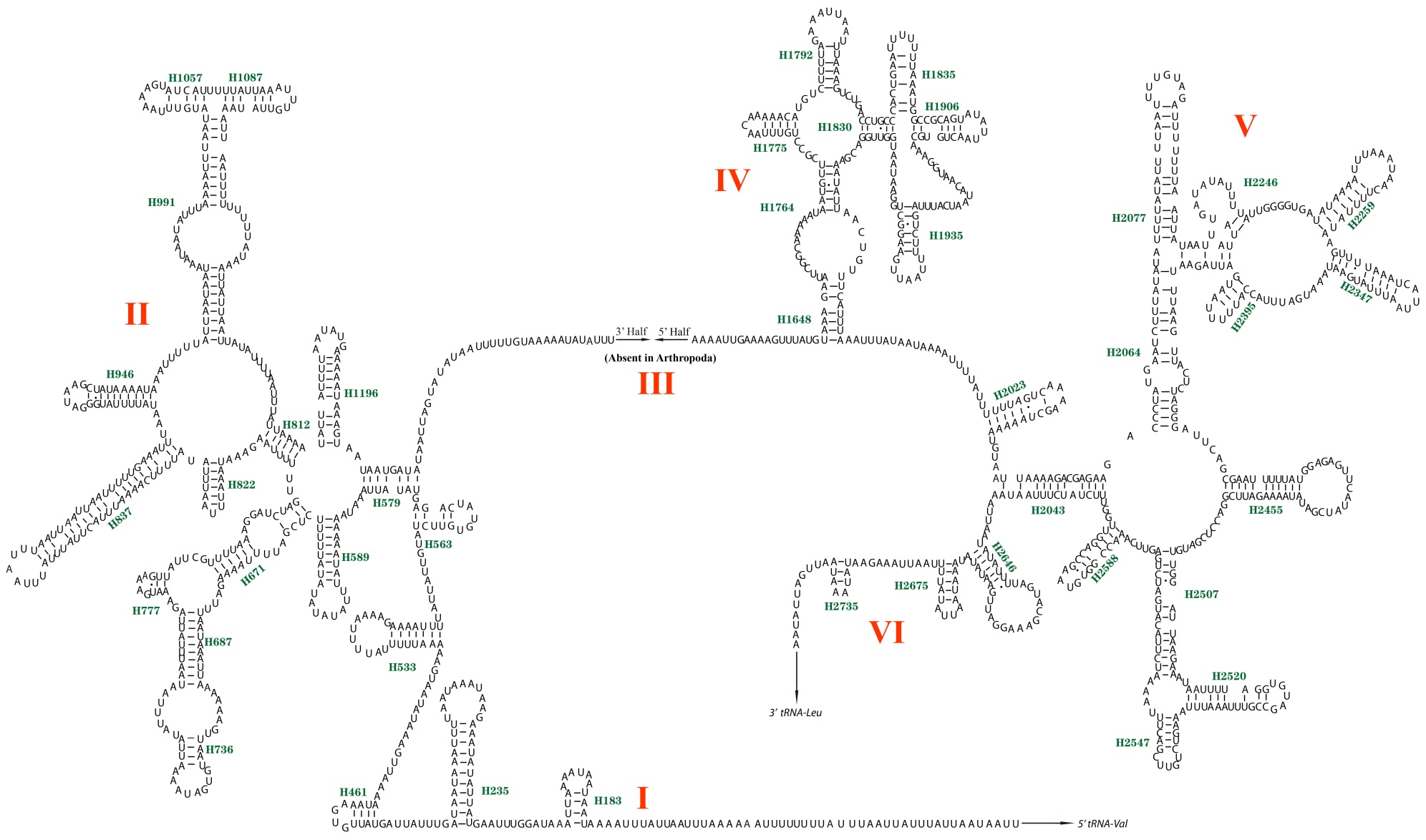


**Figure S3.** Predicted secondary structure for *16S* rRNA of *E. quinquestriatus*. The names of helices are shown in green “H+numbers.”


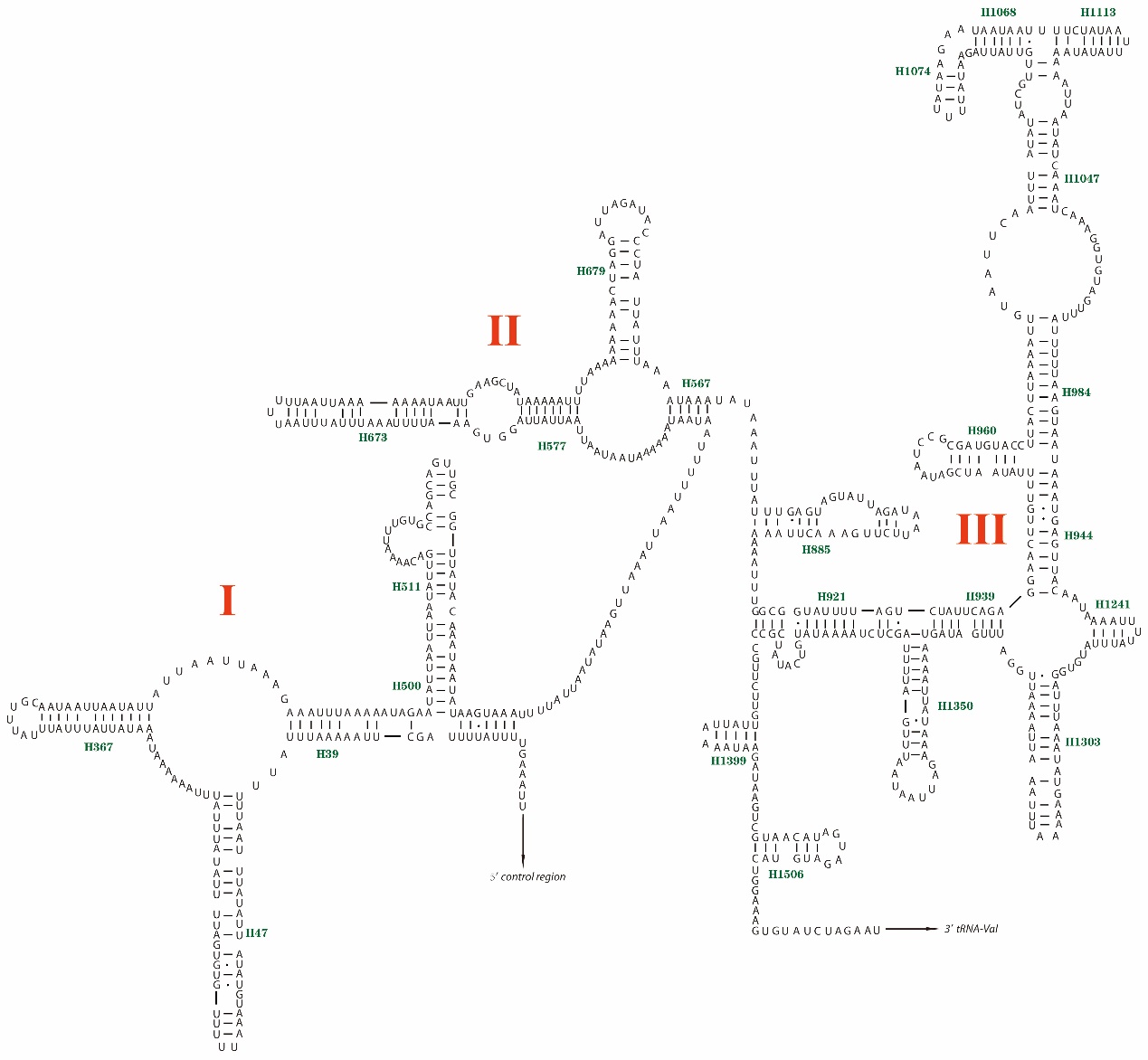


**Figure S4.** Predicted secondary structure for *12S* rRNA *E. quinquestriatus*. The names of helices are shown in green “H+numbers.”


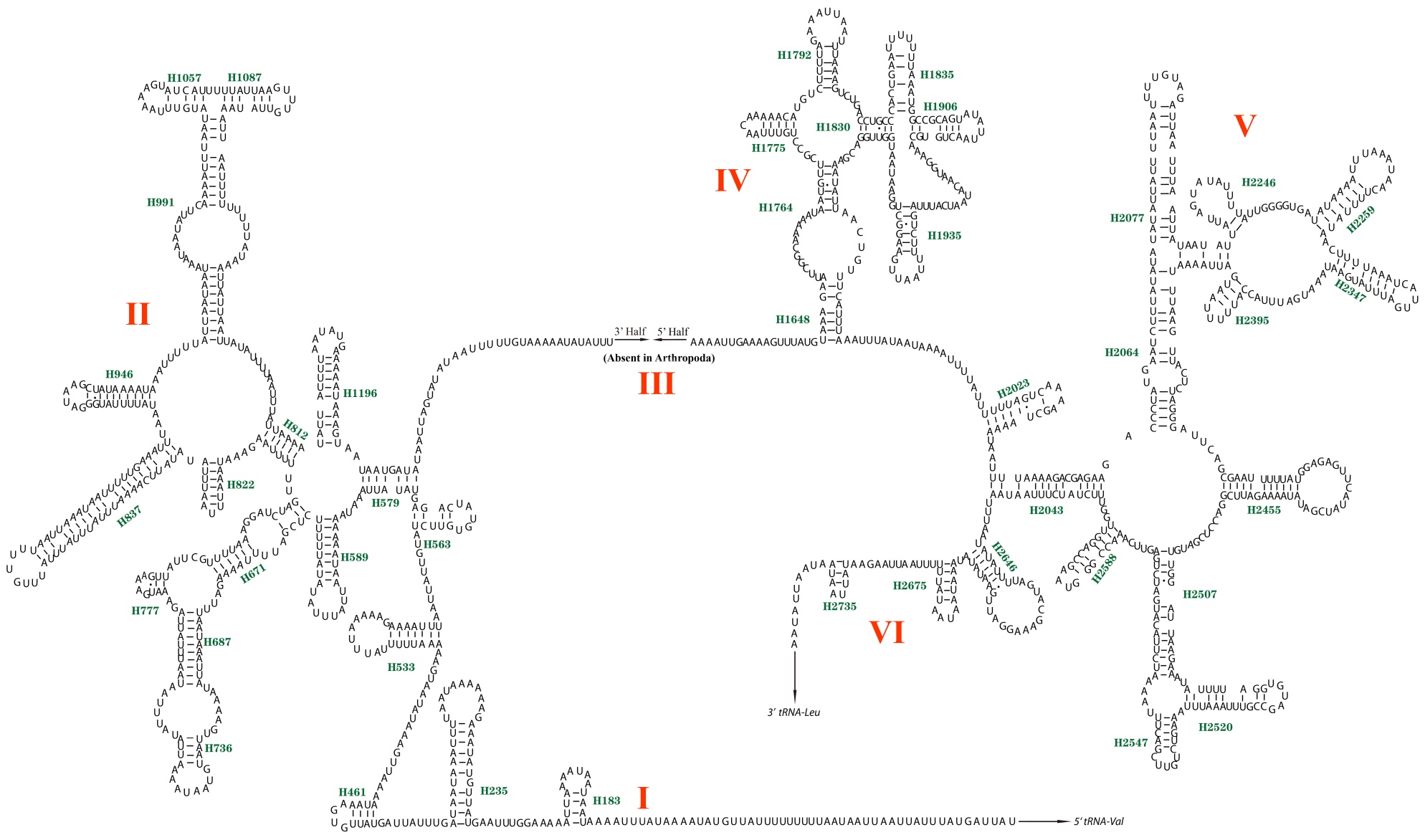


**Figure S5.** Predicted secondary structure for *16S* rRNA of *E. tarsalis*. The names of helices are shown in green “H+numbers.”


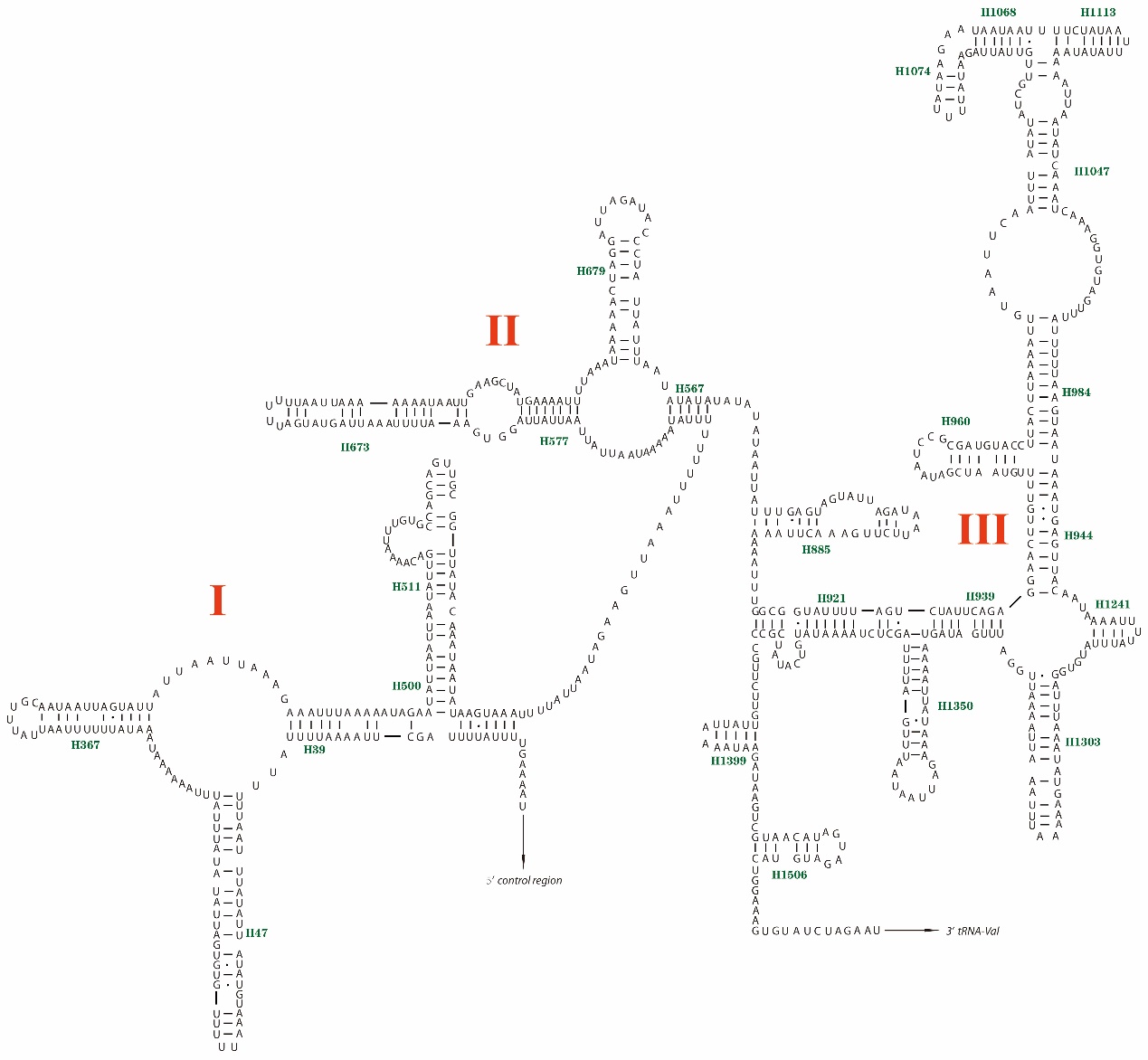


**Figure S6.** Predicted secondary structure for *12S* rRNA of *E. tarsalis*. The names of helices are shown in green “H+numbers.”


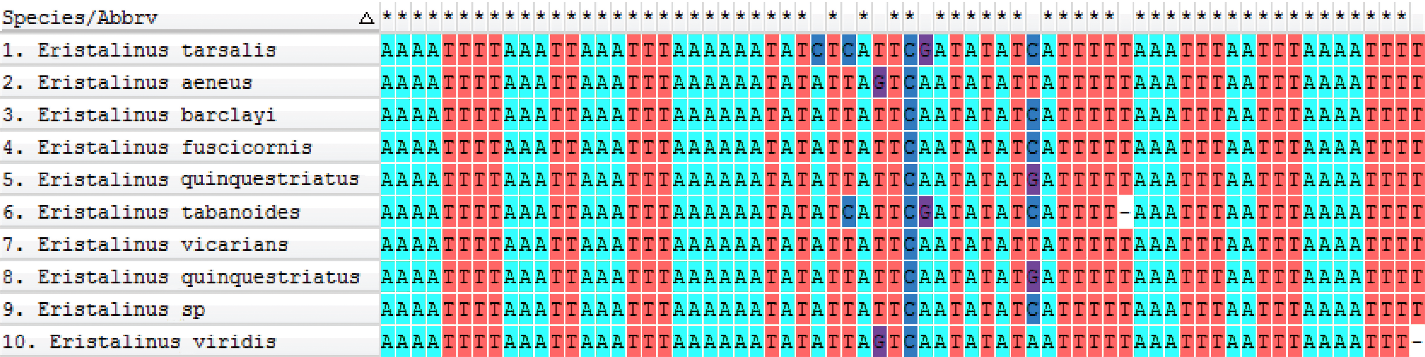


**Figure S7.** Conserved sequences of seven control regions from nine species of *Eristalinus*. *Eriatalinus* *quinquestriatus* of number 5 is from GenBank (MT834869), and of number 8 is from this study.


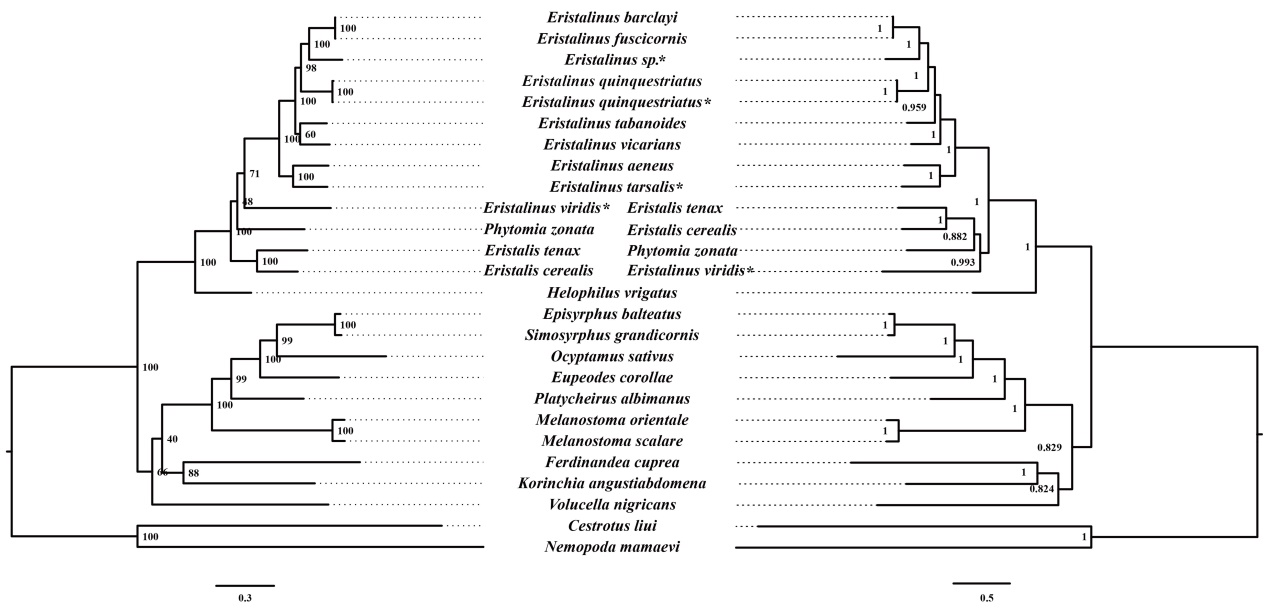


**Figure S8.** Phylogenetic trees of Syrphidae based on the PCG123 dataset using maximum likelihood (ML, left) and Bayesian inference (BI, right) methods. “Bootstrap support” (ML) and “posterior probabilities” (BI) are indicated at their nodes respectively.


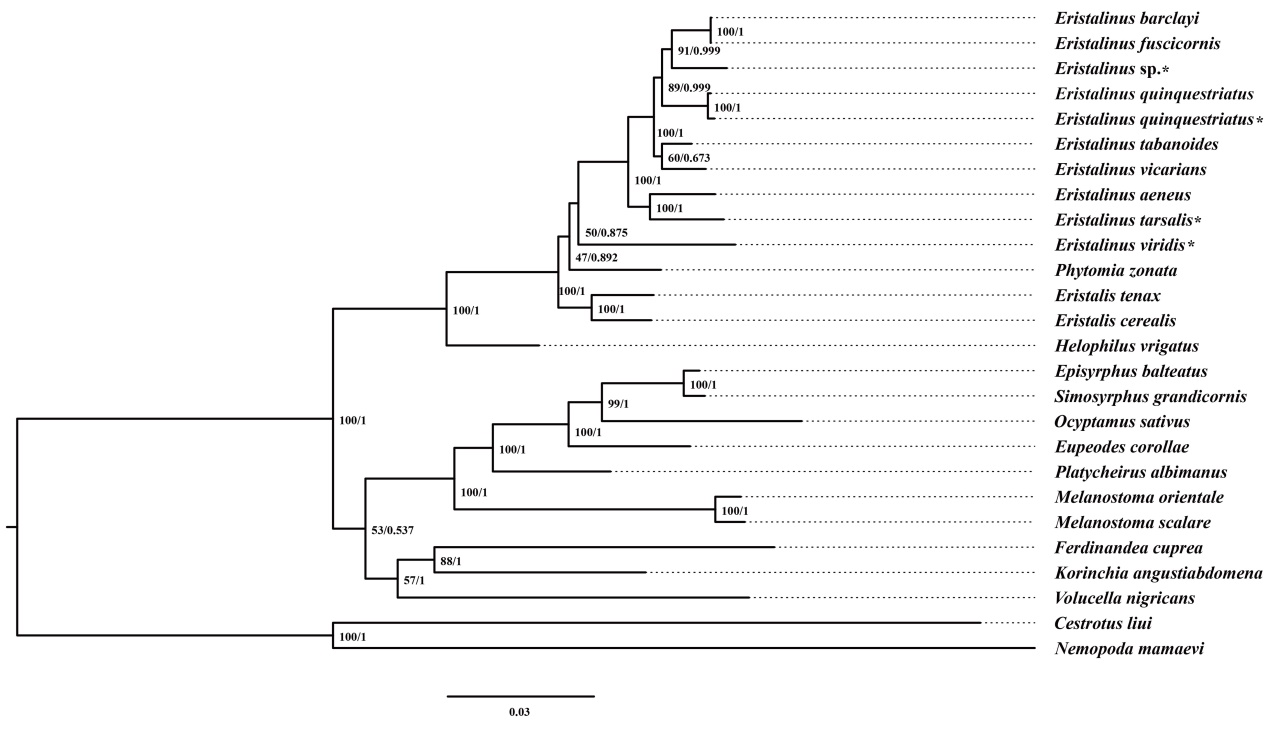


**Figure S9.** Phylogenetic tree of Syrphidae based on the PCG12RNA dataset using maximum likelihood (ML) and Bayesian inference (BI) methods. “Bootstrap support” (ML) and “posterior probabilities” (BI) are indicated at their nodes respectively.


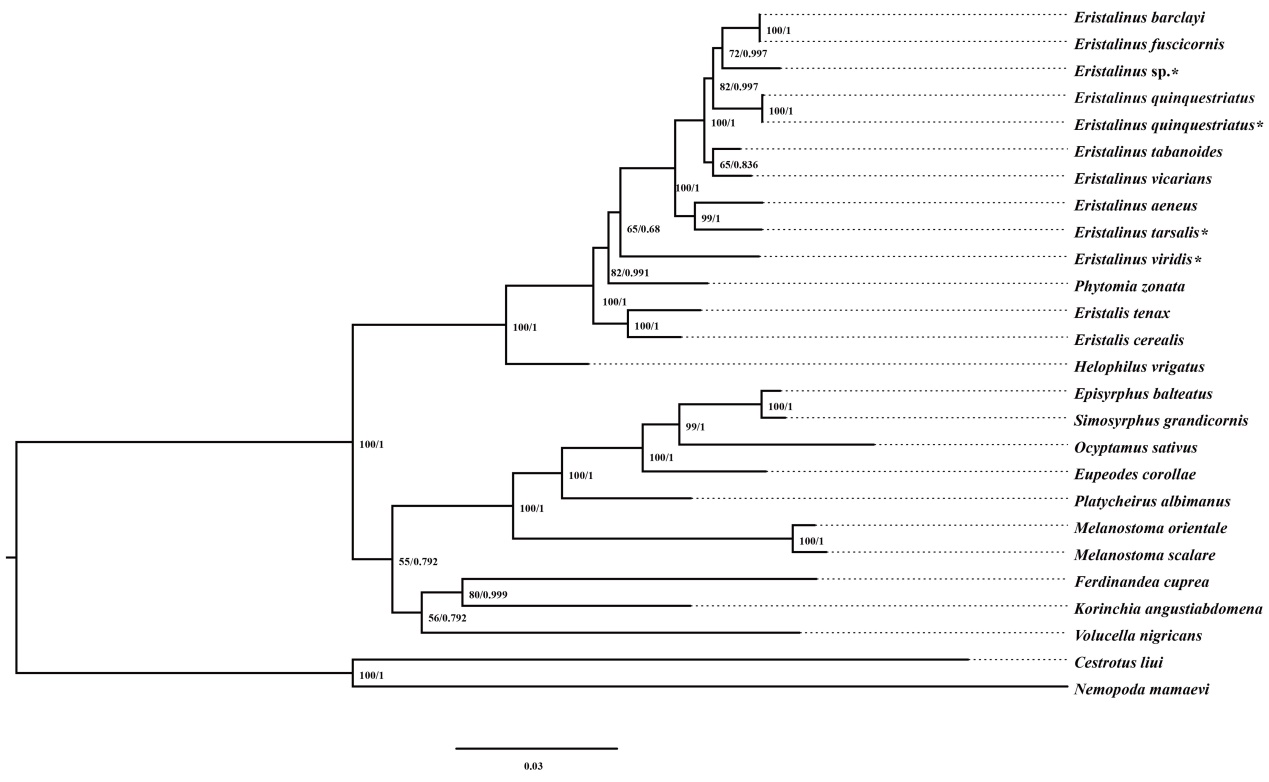


**Figure S10.** Phylogenetic tree of Syrphidae based on the PCG12 dataset using maximum likelihood (ML) and Bayesian inference (BI) methods. “Bootstrap support” (ML) and “posterior probabilities” (BI) are indicated at their nodes respectively.


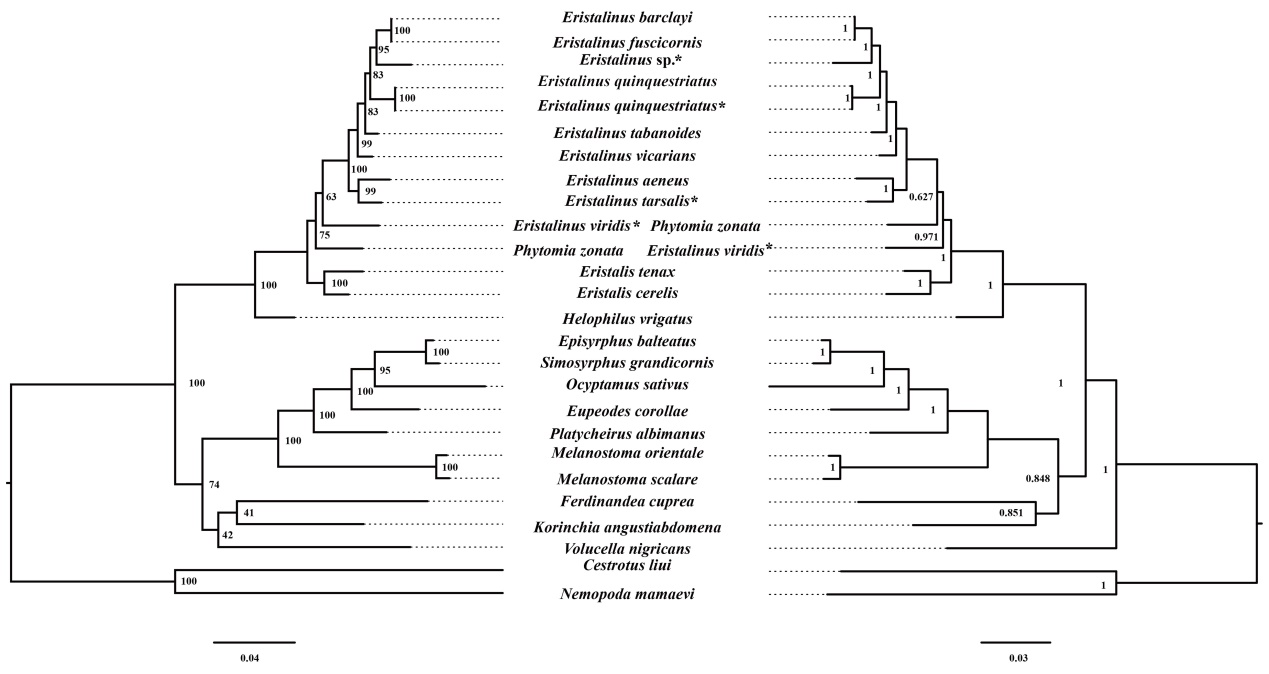


**Figure S11.** Phylogenetic trees of Syrphidae based on the AA dataset using maximum likelihood (ML, left) and Bayesian inference (BI, right) methods. “Bootstrap support” (ML) and “posterior probabilities” (BI) are indicated at their nodes respectively.
