# Supplementary material for: Comparative analysis of four complete mitogenomes from hoverfly genus Eristalinus with phylogenetic implications
Source: Sci Rep. 2022 Mar 9;12:4164. doi: 10.1038/s41598-022-08172-6 (PMC8907203; doi:10.1038/s41598-022-08172-6)
Supplement: Supplementary file 2 — Supplementary Information 2. [file 41598_2022_8172_MOESM2_ESM.docx]

Supplementary Tables

**Table S1 Collection information of the four sequenced species of *Eristalinus* (Diptera: Syrphidae) used in this study.**

| Species | Region | Latitude and longitude | Collection date | Collector |
| --- | --- | --- | --- | --- |
| *Eristalinus viridis* | Changqing National Nature Reserve, Shaanxi | 107°58′23″E, 33°35′ 48″N | 22, VII, 2018 | Hu Li |
| *Eristalinus quinquestriatus* | Changqing National Nature Reserve, Shaanxi | 107°58′23″E, 33°35′ 48″N | 22, VII, 2018 | Hu Li |
| *Eristalinus* sp. | Yingpan, Liuba, Hanzhong, Shaanxi | 106°43'20"E  33°37'2"N | 6, VIII, 2019 | Juan Li |
| *Eristalinus tarsalis* | Changqing National Nature Reserve, Shaanxi | 107°32'20.76"E  33°35'25.01"N | 10, VIII, 2019 | Juan Li |

**Table S2 The primers of *COX1* used in the study.**

| Primers name | Oligo Name | Sequence (5' to 3') |
| --- | --- | --- |
| 2312045196 | LCO1490 | ggt caa caa atc ata aag ata ttg g |
| 2312045197 | HCO2198 | taa act tca ggg tga cca aaa aat ca |

**Table S3 Partition strategies and evolutionary models used in Phylogenetic analysis.**

| Dataset | Subset | Best Model | sites | Partition names |
| --- | --- | --- | --- | --- |
| PCGRNA  14 partitions | 1 | GTR+I+G | 710 | ATP6_pos1, COX3_pos1, COX2_pos1 |
|  | 2 | TVM+I+G | 2150 | CYTB_pos2, ND1_pos2, ND3_pos2, ATP6_pos2, ND5_pos2, ND4L_pos2, ND4_pos2 |
|  | 3 | HKY+I+G | 1555 | COX3_pos3, COX1_pos3, COX2_pos3, ATP6_pos3, ND2_pos3 |
|  | 4 | GTR+I+G | 736 | ND3_pos1, ND6_pos1, ND2_pos1, ATP8_pos1, ATP8_pos3 |
|  | 5 | TVM+I+G | 566 | ND2_pos2, ATP8_pos2, ND6_pos2 |
|  | 6 | GTR+I+G | 506 | COX1_pos1 |
|  | 7 | TVM+I+G | 992 | COX3_pos2, COX2_pos2, COX1_pos2 |
|  | 8 | GTR+R | 378 | CYTB_pos1 |
|  | 9 | GTR+G | 669 | CYTB_pos3, ND6_pos3, ND3_pos3 |
|  | 10 | GTR+I+G | 1431 | ND1_pos1, ND5_pos1, ND4L_pos1, ND4_pos1 |
|  | 11 | TRN+G | 311 | ND1_pos3 |
|  | 12 | TIM+I+G | 1120 | ND4L_pos3, ND5_pos3, ND4_pos3 |
|  | 13 | GTR+I+G | 767 | s-rRNA |
|  | 14 | GTR+I+G | 1317 | l-rRNA |
| PCG123  13 partitions | 1 | GTR+I+G | 710 | ATP6_pos1, COX3_pos1, COX2_pos1 |
|  | 2 | TVM+I+G | 2150 | ND5_pos2, ND3_pos2, ATP6_pos2, ND4L_pos2, ND4_pos2, CYTB_pos2, ND1_pos2 |
|  | 3 | HKY+I+G | 1555 | COX2_pos3, ATP6_pos3, COX3_pos3, ND2_pos3, COX1_pos3 |
|  | 4 | TIM+I+G | 445 | ND2_pos1, ATP8_pos1, ATP8_pos3 |
|  | 5 | TVM+I+G | 566 | ND2_pos2, ATP8_pos2, ND6_pos2 |
|  | 6 | GTR+I+G | 506 | COX1_pos1 |
|  | 7 | TVM+I+G | 992 | COX3_pos2, COX2_pos2, COX1_pos2 |
|  | 8 | GTR+I+G | 378 | CYTB_pos1 |
|  | 9 | GTR+G | 669 | CYTB_pos3, ND3_pos3, ND6_pos3 |
|  | 10 | GTR+I+G | 1431 | ND1_pos1, ND5_pos1, ND4L_pos1, ND4_pos1 |
|  | 11 | TRN+G | 311 | ND1_pos3 |
|  | 12 | GTR+I+G | 291 | ND3_pos1, ND6_pos1 |
|  | 13 | TRN+I+G | 1120 | ND4L_pos3, ND5_pos3, ND4_pos3 |
| PCG12  8 partitions | 1 | GTR+I+G | 710 | ATP6_pos1, COX2_pos1, COX3_pos1 |
|  | 2 | TVM+I+G | 2150 | CYTB_pos2, ND1_pos2, ATP6_pos2, ND3_pos2, ND5_pos2, ND4L_pos2, ND4_pos2 |
|  | 3 | GTR+I+G | 683 | ND6_pos1, ATP8_pos1, ND2_pos1, ND3_pos1 |
|  | 4 | TVM+I+G | 566 | ND2_pos2, ND6_pos2, ATP8_pos2 |
|  | 5 | GTR+I+G | 506 | COX1_pos1 |
|  | 6 | GTR+I+G | 992 | COX3_pos2, COX2_pos2, COX1_pos2 |
|  | 7 | GTR+G | 380 | CYTB_pos1 |
|  | 8 | GTR+I+G | 1429 | ND1_pos1, ND5_pos1, ND4L_pos1, ND4_pos1 |
| PCG12RNA  10 partitions | 1 | GTR+I+G | 710 | ATP6_pos1, COX3_pos1, COX2_pos1 |
|  | 2 | TVM+I+G | 2150 | CYTB_pos2, ND1_pos2, ATP6_pos2, ND3_pos2, ND5_pos2, ND4L_pos2, ND4_pos2 |
|  | 3 | GTR+I+G | 683 | ND3_pos1, ND6_pos1, ND2_pos1, ATP8_pos1 |
|  | 4 | TVM+I+G | 566 | ND2_pos2, ND6_pos2, ATP8_pos2 |
|  | 5 | GTR+I+G | 506 | COX1_pos1 |
|  | 6 | TVM+I+G | 992 | COX3_pos2, COX2_pos2, COX1_pos2 |
|  | 7 | GTR+G | 380 | CYTB_pos1 |
|  | 8 | GTR+I+G | 1429 | ND1_pos1, ND5_pos1, ND4L_pos1, ND4_pos1 |
|  | 9 | GTR+I+G | 767 | s-rRNA |
|  | 10 | GTR+I+G | 1317 | l-rRNA |

**Table S4 Complete mitochondrial genome organization of *Eristalinus viridis*.**

| Name | Direction | Location | Size(bp) | Anticodon | Start/stop codon | Intergenic Sequence (bp) |
| --- | --- | --- | --- | --- | --- | --- |
| *tRNA*-*I* | F | 1-65 | 65 | 30-32/GAT |  | 0 |
| *tRNA*-*Q* | R | 63-131 | 69 | 101-99/TTG |  | -3 |
| *tRNA*-*M* | F | 136-204 | 69 | 166-168/CAT |  | 4 |
| *ND2* | F | 205-1227 | 1023 |  | ATT/TAA | 0 |
| *tRNA*-*W* | F | 1225-1294 | 70 | 1256-1258TCA |  | -3 |
| *tRNA*-*C* | R | 1286-1352 | 67 | 1323-1321/GCA |  | -9 |
| *tRNA*-*Y* | R | 1354-1420 | 67 | 1389-1387/GTA |  | 1 |
| *COX1* | F | 1521-2957 | 1437 |  | ATT/TAA | 0 |
| *tRNA*-*L1* | F | 2953-3018 | 66 | 2982-2984/TAA |  | -5 |
| *COX2* | F | 3023-3706 | 684 |  | ATG/TAA | 4 |
| *tRNA*-*K* | F | 3708-3778 | 71 | 3738-3740/CTT |  | 1 |
| *tRNA*-*D* | F | 3781-3847 | 67 | 3812-3814/GTC |  | 2 |
| *ATP8* | F | 3845-4009 | 165 |  | TTG/TAA | -3 |
| *ATP6* | F | 3997-4680 | 684 |  | TTG/TAA | -13 |
| *COX3* | F | 4681-5475 | 795 |  | ATT/TAA | 0 |
| *tRNA*-*G* | F | 5479-5548 | 70 | 5508-5510/TCC |  | 3 |
| *ND3* | F | 5546-5902 | 357 |  | ATA/TAG | 3 |
| *tRNA*-*A* | F | 5901-5965 | 65 | 5932-5934/TGC |  | -2 |
| *tRNA*-*R* | F | 5968-6030 | 63 | 5997-5999/TCG |  | 2 |
| *tRNA*-*N* | F | 6033-6100 | 68 | 6064-6066/GTT |  | 2 |
| *tRNA*-*S1* | F | 6101-6167 | 67 | 6126-6128/GCT |  | 0 |
| *tRNA*-*E* | F | 6168-6233 | 66 | 6198-6200/TTC |  | 0 |
| *tRNA*-*F* | R | 6263-6329 | 67 | 6297-6295/GAA |  | 29 |
| *ND5* | R | 6330-8064 | 1735 |  | ATT/T-- | 0 |
| *tRNA*-*H* | R | 8062-8128 | 67 | 8097-8095/GTG |  | -3 |
| *ND4* | R | 8129-9469 | 1341 |  | ATG/TAA | 0 |
| *ND4L* | R | 9463-9759 | 297 |  | ATG/TAA | -7 |
| *tRNA*-*T* | F | 9762-9827 | 66 | 9792-9794/TGT |  | 2 |
| *tRNA*-*P* | R | 9828-9893 | 66 | 9863-9861/TGG |  | 0 |
| *ND6* | F | 9896-10420 | 525 |  | ATT/TAA | 2 |
| *Cytb* | F | 10420-11556 | 1137 |  | ATG/TAA | -1 |
| *tRNA*-*S2* | F | 11562-11629 | 68 | 11591-11593/TGA |  | 5 |
| *ND1* | R | 11651-12592 | 942 |  | TTG/TAG | 21 |
| *tRNA*-*L2* | R | 12594-12658 | 65 | 12629-12627/TAG |  | 1 |
| *16S* | R | 12659-13991 | 1333 |  |  | 0 |
| *tRNA*-*V* | R | 13992-14063 | 72 | 14030-14028/TAC |  | 0 |
| *12S* | R | 14064-14856 | 793 |  |  | 0 |
| CR | F | 14857-15640 | 784 |  |  | 0 |

**Table S5 Complete mitochondrial genome organization of *Eristalinus quinquestriatus*.**

| Name | Direction | Location | Size (bp) | Anticodon | Start/stop codon | Intergenic Sequence (bp) |
| --- | --- | --- | --- | --- | --- | --- |
| *tRNA*-*I* | F | 1-66 | 66 | 28-30/TTG |  | 0 |
| *tRNA*-*Q* | R | 64-132 | 69 | 102-100/TTG |  | -3 |
| *tRNA*-*M* | F | 137-205 | 69 | 167-139/CAT |  | 4 |
| *ND2* | F | 206-1228 | 1023 |  | ATT/TAA | 0 |
| *tRNA*-*W* | F | 1226-1295 | 70 | 1257-1259/TCA |  | -3 |
| *tRNA*-*C* | R | 1287-1355 | 69 | 1326-1324/GCA |  | -9 |
| *tRNA*-*Y* | R | 1378-1444 | 67 | 1413-1411/GTA |  | 22 |
| *COX1* | F | 1479-2981 | 1503 |  | ATT/TAA | 34 |
| *tRNA*-*L1* | F | 2977-3042 | 66 | 3006-3008/TAA |  | -5 |
| *COX2* | F | 3049-3732 | 684 |  | ATG/TAA | 6 |
| *tRNA*-*K* | F | 3734-3804 | 71 | 3764-3766/CTT |  | 1 |
| *tRNA*-*D* | F | 3816-3882 | 67 | 3847-3849/GTC |  | 11 |
| *ATP8* | F | 3883-4033 | 151 |  | TTG/TAA | 0 |
| *ATP6* | F | 4032-4715 | 684 |  | TTG/TAA | -2 |
| *COX3* | F | 4721-5509 | 789 |  | ATT/TAA | 5 |
| *tRNA*-*G* | F | 5513-5580 | 68 | 5542-5544/TCC |  | 3 |
| *ND3* | F | 5578-5934 | 357 |  | ATA/TAG | -3 |
| *tRNA*-*A* | F | 5933-6003 | 71 | 5967-5969TGC |  | -2 |
| *tRNA*-*R* | F | 6003-6065 | 63 | 6032-6034/TCG |  | -1 |
| *tRNA*-*N* | F | 6081-6147 | 67 | 6112-6114/GTT |  | 15 |
| *tRNA*-*S1* | F | 6148-6214 | 67 | 6173-6175/GCT |  | 0 |
| *tRNA*-*E* | F | 6215-6282 | 68 | 6247-6249/TTC |  | 0 |
| *tRNA*-*F* | R | 6315-6381 | 67 | 6349-6347/GAA |  | 32 |
| *ND5* | R | 6382-8116 | 1735 |  | ATT/T-- | 0 |
| *tRNA*-*H* | R | 8114-8179 | 66 | 8147-8149/GTG |  | -3 |
| *ND4* | R | 8179-9519 | 1341 |  | ATG/TAA | -1 |
| *ND4L* | R | 9513-9809 | 297 |  | ATG/TAA | -7 |
| *tRNA*-*T* | F | 9812-9877 | 66 | 9842-9844/TGT |  | 2 |
| *tRNA*-*P* | R | 9878-9944 | 67 | 9913-9911/TGG |  | 0 |
| *ND6* | F | 9947-10471 | 525 |  | ATT/TAA | 2 |
| *Cytb* | F | 10471-11607 | 1137 |  | ATG/TAA | -1 |
| *tRNA*-*S2* | F | 11615-11682 | 68 | 11644-11646/TGA |  | 7 |
| *ND1* | R | 11704-12645 | 942 |  | TTG/TAG | 21 |
| *tRNA*-*L2* | R | 12646-12711 | 66 | 12682-12680/TAG |  | 0 |
| *16S* | R | 12712-14048 | 1337 |  |  | 0 |
| *tRNA*-*V* | R | 14049-14120 | 72 | 14087-14085/TAC |  | 0 |
| *12S* | R | 14121-14913 | 793 |  |  | 0 |
| CR | F | 14914-15872 | 959 |  |  | 0 |

**Table S6 Complete mitochondrial genome organization of *Eristalinus* sp.**

| Name | Direction | Location | Size(bp) | Anticodon | start/stop codon | Intergenic Sequence (bp) |
| --- | --- | --- | --- | --- | --- | --- |
| *tRNA*-*I* | F | 1-66 | 66 | 30-32/GAT |  | 0 |
| *tRNA*-*Q* | R | 64-132 | 69 | 93-91/TTG |  | -3 |
| *tRNA*-*M* | F | 138-206 | 69 | 168-170/CAT |  | 5 |
| *ND2* | F | 207-1229 | 1023 |  | ATC/TAA | 0 |
| *tRNA*-*W* | F | 1227-1296 | 70 | 1258-1260/TCA |  | -3 |
| *tRNA*-*C* | R | 1288-1356 | 69 | 1327-1325/GCA |  | 9 |
| *tRNA*-*Y* | R | 1370-1436 | 67 | 1405-1403/GTA |  | 13 |
| *COX1* | F | 1471-2973 | 1503 |  | ATT/TAA | 34 |
| *tRNA*-*L1* | F | 2969-3034 | 66 | 2998-3000/TAA |  | -5 |
| *COX2* | F | 3036-3725 | 690 |  | ATT/TAA | 0 |
| *tRNA*-*K* | F | 3727-3797 | 71 | 3757-3759/CTT |  | 1 |
| *tRNA*-*D* | F | 3808-3874 | 67 | 3839-3841/GTC |  | 10 |
| *ATP8* | F | 3875-4036 | 162 |  | ATC/TAA | 0 |
| *ATP6* | F | 4033-4707 | 675 |  | ATA/TAA | -4 |
| *COX3* | F | 4709-5497 | 789 |  | ATG/TAA | 1 |
| *tRNA*-*G* | F | 5501-5567 | 67 | 5530-5532/TCC |  | 3 |
| *ND3* | F | 5568-5921 | 354 |  | ATT/TAG | 0 |
| *tRNA*-*A* | F | 5919-5990 | 72 | 5953-5955/TGC |  | -3 |
| *tRNA*-*R* | F | 5989-6054 | 66 | 6018-6020/TCG |  | -2 |
| *tRNA*-*N* | F | 6080-6146 | 67 | 6111-6113/GTT |  | 25 |
| *tRNA*-*S1* | F | 6147-6213 | 67 | 6172-6174/GCT |  | 0 |
| *tRNA*-*E* | F | 6214-6282 | 69 | 6246-6248/TTC |  | 0 |
| *tRNA*-*F* | R | 6314-6381 | 68 | 6348-6346/GAA |  | 31 |
| *ND5* | R | 6382-8113 | 1732 |  | ATT/T-- | 0 |
| *tRNA*-*H* | R | 8114-8179 | 66 | 8149-8147/GTG |  | 0 |
| *ND4* | R | 8179-9519 | 1341 |  | ATG/TAA | -1 |
| *ND4L* | R | 9513-9809 | 297 |  | ATG/TAA | -7 |
| *tRNA*-*T* | F | 9812-9877 | 66 | 9842-9844/TGT |  | 2 |
| *tRNA*-*P* | R | 9878-9944 | 67 | 9913-9911/TGG |  | 0 |
| *ND6* | F | 9947-10471 | 525 |  | ATT/TAA | 2 |
| *Cytb* | F | 10471-11607 | 1137 |  | ATG/TAA | -1 |
| *tRNA*-*S2* | F | 11617-11684 | 68 | 11646-11648/TGA |  | 9 |
| *ND1* | R | 11706-12647 | 942 |  | TTG/TAA | 21 |
| *tRNA*-*L2* | R | 12649-12713 | 65 | 12684-12682/TAG |  | 1 |
| *16S* | R | 12714-14059 | 1346 |  |  | 0 |
| *tRNA*-*V* | R | 14060-14130 | 71 | 14098-14096/TAC |  | 0 |
| *12S* | R | 14131-14923 | 793 |  |  | 0 |
| CR | F | 14924-15883 | 960 |  |  | 0 |

**Table S7 Complete mitochondrial genome organization of *Eristalinus tarsalis*.**

| Name | Direction | Location | Size(bp) | Anticode | start/stop codon | Intergenic Sequence (bp) |
| --- | --- | --- | --- | --- | --- | --- |
| *tRNA*-*I* | F | 1-66 | 66 | 30-32/GAT |  | 0 |
| *tRNA*-*Q* | R | 85-153 | 69 | 123-121/TTG |  | 18 |
| *tRNA*-*M* | F | 164-232 | 69 | 194-196/CAT |  | 10 |
| *ND2* | F | 233-1255 | 1023 |  | ATC/TAA | 0 |
| *tRNA*-*W* | F | 1253-1322 | 70 | 1284-1286/TCA |  | -3 |
| *tRNA*-*C* | R | 1329-1398 | 70 | 1369-1367/GCA |  | 6 |
| *tRNA*-*Y* | R | 1407-1473 | 67 | 1440-1442/GTA |  | 8 |
| *COX1* | F | 1508-3010 | 1503 |  | ATT/TAA | 14 |
| *tRNA*-*L1* | F | 3006-3071 | 66 | 3035-3037/TAA |  | -5 |
| *COX2* | F | 3076-3759 | 684 |  | ATG/TAA | 4 |
| *tRNA*-*K* | F | 3761-3831 | 71 | 3791-3793/CTT |  | 0 |
| *tRNA*-*D* | F | 3846-3912 | 67 | 3877-3879/GTC |  | 14 |
| *ATP8* | F | 3913-4074 | 162 |  | ATT/TAA | 0 |
| *ATP6* | F | 4071-4745 | 675 |  | ATA/TAA | -4 |
| *COX3* | F | 4759-5547 | 789 |  | ATG/TAA | 13 |
| *tRNA*-*G* | F | 5551-5617 | 67 | 5580-5582/TCC |  | 3 |
| *ND3* | F | 5618-5971 | 354 |  | ATT/TAG | 0 |
| *tRNA*-*A* | F | 5980-6049 | 70 | 6013-6015/TGC |  | 8 |
| *tRNA*-*R* | F | 6049-6112 | 64 | 6079-6081/TCG |  | -1 |
| *tRNA*-*N* | F | 6124-6190 | 67 | 6155-6157/GTT |  | 11 |
| *tRNA*-*S1* | F | 6191-6257 | 67 | 6216-6218/GCT |  | 0 |
| *tRNA*-*E* | F | 6261-6327 | 67 | 6292-6294/TCA |  | 3 |
| *tRNA*-*F* | R | 6364-6431 | 68 | 6398-6396/GAA |  | 36 |
| *ND5* | R | 6432-8166 | 1735 |  | ATT/T-- | 0 |
| *tRNA*-*H* | R | 8164-8229 | 66 | 8199-8197/GTG |  | -3 |
| *ND4* | R | 8236-9576 | 1341 |  | ATG/TAA | 6 |
| *ND4L* | R | 9570-9866 | 297 |  | ATG/TAA | -7 |
| *tRNA*-*T* | F | 9869-9934 | 66 | 9899-9901/TGT |  | 2 |
| *tRNA*-*P* | R | 9935-10000 | 66 | 9970-9968/TGG |  | 0 |
| *ND6* | F | 10003-10527 | 525 |  | ATT/TAA | 2 |
| *Cytb* | F | 10527-11663 | 1137 |  | ATG/TAA | -1 |
| *tRNA*-*S2* | F | 11672-11739 | 68 | 11701-11703 TGA |  | 8 |
| *ND1* | R | 11761-12702 | 942 |  | TTG/ TAG | 21 |
| *tRNA*-*L2* | R | 12704-12768 | 65 | 12739-12737/TAG |  | 1 |
| *16S* | R | 12769-14104 | 1336 |  |  | 0 |
| *tRNA*-*V* | R | 14105-14176 | 72 | 14143-14141/TAC |  | 0 |
| *12S* | R | 14177-14966 | 790 |  |  | 0 |
| CR | F | 14967-15849 | 883 |  |  | 0 |
